# Enhanced immobilization of cadmium, lead, and antimony with improved soil fertility using sulfate-reducing bacteria@nano zero-valent iron-modified biochar: coupled chemisorption and microbial mechanisms

**DOI:** 10.3389/fmicb.2025.1712696

**Published:** 2026-01-05

**Authors:** Shirui Peng, Fengshuo Ya, Juan Yin, Changjun Liao, Dangling Qin, Jiapan Lian, Hong Li, Hailong Wang, Jianming Xue, Xiaoe Yang, Hongfei Lin, Jiancheng Chen, Guofei Pan, Yanyan Wei

**Affiliations:** 1 State Key Laboratory for Conservation and Utilization of Subtropical Agri-Bioresources, Guangxi Key Laboratory for Agro-Environment and Agro-Products Safety, National Demonstration Center for Experimental Plant Science Education, College of Agriculture, Guangxi University, Nanning, China; 2 Bossco Environmental Protection Technology Co., Ltd., Nanning, China; 3 Key Laboratory of Eco-Environment of Three Gorges Region, Ministry of Education, Chongqing University, Chongqing, China; 4 School of Environment and Chemical Engineering, Foshan University, Foshan, China; 5 New Zealand Forest Research Institute (Scion), Forest System, Christchurch, New Zealand; 6 Ministry of Education Key Laboratory of Environmental Remediation and Ecological Health, College of Environmental and Resource Sciences, Zhejiang University, Hangzhou, China

**Keywords:** immobilized bacteria, heavy metal, biochar, nanoscale zero valent iron, SRB

## Abstract

**Introduction:**

Soil co-contamination with cadmium (Cd), lead (Pb), and antimony (Sb) poses significant environmental and health risks, highlighting the need for effective remediation strategies. Sulfate-reducing bacteria (SRB) are promising for bioremediation, but require optimization to improve effectiveness.

**Methods:**

Here, we developed SRB@nZVI@BC, a novel composite integrating SRB, nano zero-valent iron-modified biochar (nZVI@BC), and sodium alginate (SA). Its optimal preparation conditions were identified as 2% SA, 2% CaCl2, 30% SRB solution, and 0.1% nZVI@BC based on mass transfer performance, mechanical strength, and sulfate reduction rate.

**Results:**

The application of SRB@nZVI@BC increased the proportion of stable forms of Cd, Pb, and Sb in soil and achieved removal efficiencies of 60.22%–63.93% for Cd, 57.13%–59.45% for Pb, and 56.02%–70.37% for Sb in leachate. Compared to alone SRB treatment, SRB@nZVI@BC significantly enhanced SRB activity, promoting sulfur cycling and the generation of S^2−^, thereby facilitated heavy metal precipitation as insoluble sulfides. SRB@nZVI@BC could improve the adsorption capacity of soil for heavy metals by activating the oxygen-containing functional groups such as C-O-C. Moreover, SRB@nZVI@BC reshaped the soil microbial community by enriching sulfate-reducing genera such as *Desulfosporosinus* and *Desulfitobacterium*, driving heavy metal transformation and stabilization. The composite further enhanced soil nutrient availability (N, P, K) and increased enzyme activities, contributing to soil fertility recovery.

**Discussion:**

Overall, SRB@nZVI@BC provides an eco-friendly solution for stabilizing multi-metal-contaminated soils and promoting the restoration of barren lands through synergistic adsorption and biomineralization.

## Graphical abstract

**Figure:**
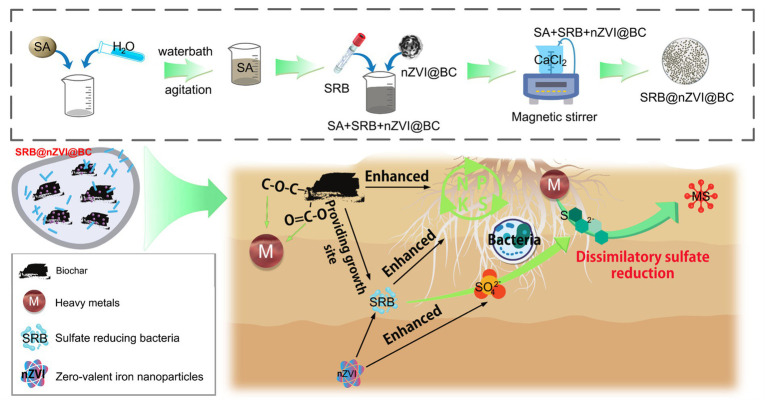

## Highlights

- SRB@nZVI@BC immobilizes Cd, Pb, and Sb by facilitating their precipitation as insoluble sulfides.
- SRB@nZVI@BC improved soil Cd, Pb, and Sb adsorption via functional groups (C–O–C, O=C–O and Fe–O).
- SRB@nZVI@BC reshaped the soil microbial community, especially enriching sulfate-reducing genera.

## 1 Introduction

Heavy metal pollution has garnered significant attention due to its non-degradable characteristics. A 2014 National Soil Pollution Survey revealed that 19.4% of China’s arable land exceeded soil quality standards. Among the pollutants, cadmium (Cd) and lead (Pb) exceedance rates had reached 7.0 and 1.5%, respectively (Zeng et al., 2019). These contaminants degrade soil quality, disrupt microbial activity, harm plant growth, and pose long-term risks to human health. Therefore, low-cost, effective, and eco-friendly remediation technologies are urgently needed.

Various methods, including physicochemical and phytoremediation approaches, have been applied to remediate heavy metal-contaminated soils. However, each has notable drawbacks. Chemical remediation, though effective, is expensive, disrupts soil structure, requires large quantities of reagents, and may cause secondary pollution (Xu et al., 2023). Phytoremediation is limited by long treatment periods, uncertain effectiveness, and poor adaptability of plants in diverse environmental conditions (Wei et al., 2025). To overcome these limitations, more sustainable and efficient technologies are being explored.

Microbial remediation technology has gained prominence in soil heavy metal remediation due to its low cost, high safety, and ease of application (Mai et al., 2025). Among these, sulfate-reducing bacteria (SRB) are promising agents for heavy metal immobilization due to their ability to generate sulfide ions (S^2−^), which react with metal ions to form insoluble precipitates (Drennan et al., 2016; Hu et al., 2024). For instance, Diaz-Muniz et al. (2024) used SRB-generated HS^−^ to precipitate Pb as PbS, achieving up to 97% removal. Kiran et al. (2017) used SRB to remove a variety of heavy metals from wastewater and found that the removal of Cu, Ni, Zn, Cd, Pb, and Fe was more than 90%. According to Dong et al. (2024), SRB can significantly enhance the removal of Pb from tailings and acid mine drainage through the biogenic formation of insoluble PbS precipitates. However, most studies focus on SRB application in aqueous systems, with limited exploration of SRB mechanisms in composite-contaminated soils. Furthermore, direct application of free SRBs for soil bioremediation faces challenges from unfavorable environmental conditions (e.g., pH, electron donor availability, competition with indigenous microorganisms), hindering SRB growth and activity (Yu et al., 2020). These issues are more severe in complex soil environments, underscoring the need for advanced strategies to enhance SRB stability and performance.

Microbial immobilization enhances the stability and activity of functional microorganisms by confining them within a defined spatial structure, reducing direct exposure to toxic substances (Partovinia and Rasekh, 2018; Qin et al., 2020). Biochar is a carbon-rich material produced by the pyrolysis of organic matter under oxygen-limited conditions. Owing to its wide availability of raw materials, simple preparation process, and stable chemical properties, it has been extensively applied in the field of environmental remediation (Li Y. et al., 2024). Biochar’s loose and porous surface structure makes it an ideal inorganic carrier for microbial immobilization, providing habitat and promoting growth (Zhao et al., 2025). Biochar also reduces competition for nutrients among indigenous microorganisms and mitigates adverse environmental factors, thereby stabilizing microbial activity and growth (Ji et al., 2022). Thus, the immobilization of microorganisms onto biochar enhances remediation efficiency while improving soil structure and fertility. Zero-valent iron nanoparticles (nZVI) are strong reductants with low cost and minimal environmental impact (Zhang H. et al., 2023). During oxidation, nZVI consume oxygen and lower redox potential, thereby stimulating SRB activity. Hydrogen produced from nZVI corrosion acts as an electron donor, further enhancing SRB growth and metal reduction (Teng et al., 2020). When nZVI are loaded onto biochar, the composite exhibits significantly increased surface area and enriched functional groups, thereby enhancing its capacity to adsorb heavy metals (Li C. et al., 2024). Zhang et al. (2025) reported that the application of ferric sulfate-modified biochar (Fe-BC) reduced Cr accumulation in rice grains by 56%. Similarly, Shang et al. (2024) synthesized an iron-based pyrolyzed biochar (Fe-PEWC), which effectively decreased Se and Cr accumulation in ryegrass by 71.25 and 76.43%, respectively, thereby mitigating metal toxicity. Thus, the incorporation of synergistic iron-based materials may help create a favorable environment for SRB, thereby enhancing bioremediation efficiency.

To fabricate a porous spherical carrier integrating BC and nZVI with SRB, polymeric matrices like sodium alginate (SA) were used as embedding materials. SA, a natural polysaccharide from seaweed, forms insoluble gel materials by cross-linking with divalent metal ions like Ca^2+^ (Xiang et al., 2025). These materials are biocompatible, cost-effective, and capable of producing stable bio-beads. Based on recent advancements, we propose that a composite carrier, SRB@nZVI@BC, which combines the reductive properties of nZVI@BC and the biocompatibility of SA, holds significant potential. Additionally, application of SRB@nZVI@BC may promote the recruitment of other beneficial soil functional bacteria, thereby enhancing overall soil health. However, despite its promising potential, this approach has not yet been reported in the literature. This study aims to (1) optimize the formulations of nZVI@BC, SA, and SRB to develop a composite material for heavy metal immobilization in soil; (2) evaluate the effectiveness of SRB@nZVI@BC in simultaneously immobilizing Cd, Pb, and Sb while improving soil nutrient content; (3) investigate interactions among heavy metals, environmental factors, and bacterial community functions. This study will contribute to the development of a microbial-driven remediation strategy aimed at restoring barren soils co-contaminated with Cd, Pb, and Sb in practical applications.

## 2 Materials and methods

### 2.1 Source and cultivation of SRB

SRB (*Desulfovibrio desulfuricans* subsp. *desulfuricans*, CGMCC No. 1.3469) was obtained from the China General Microbiological Culture Collection Center. The improved Starkey medium (medium composition refer Supplementary Table S1) was prepared and filled with nitrogen for 15 min to access the SRBs for incubation. Cell morphology was characterized using scanning electron microscopy (ZEISS Sigma 300). Growth conditions were optimized by systematically varying incubation temperature, initial pH, and sulfate concentration to determine parameters that maximize biomass and sulfide production.

### 2.2 Material preparation

#### 2.2.1 Optimization of preparation conditions for immobilized SRB beads

Sodium alginate (SA) was dissolved in 100 mL of sterile water and heated to 85 °C in a water bath until fully dissolved. After cooling to room temperature, SRB culture in the logarithmic growth phase was added and mixed thoroughly. The SRB-SA mixture was then dropped into a 2% CaCl_2_ solution using a sterile syringe to form spherical beads. These beads were crosslinked at 24 °C for 2 h and stored at 4 °C for 12 h. The resulting immobilized SRB beads were washed with sterile saline and preserved at 4 °C for subsequent experiments (Bouabidi et al., 2019). A three-factor, three-level orthogonal experimental design [L9(3^4^)] was employed to optimize immobilization parameters: A—SA concentration, B—CaCl_2_ concentration, and C—SRB suspension concentration (Supplementary Table S2). Bead physical properties, including specific surface area (via BET), mechanical strength, and mass transfer characteristics (Supplementary Text S1), were evaluated. Chemical performance was assessed via SRB activity in Starkey medium by measuring time-to-blackening, oxidation–reduction potential (ORP), and sulfate consumption. Optimal preparation conditions were selected based on the combined performance of these metrics.

#### 2.2.2 Preparation of composite materials SRB@nZVI@BC

Biochar (BC) was produced by pyrolyzing rice straw at 550 °C for 2 h in a limited oxygen environment, following a ramp rate of 10 °C/min. The resulting biochar was ground and sieved through a 100-mesh screen. The nZVI@BC composite was synthesized as follows: FeSO_4_·7H_2_O solution (0.054 mol/L) was purged with N_2_ to remove dissolved oxygen. BC was then added to the solution at an impregnation mass ratio of Fe to BC (Fe:C) of 5:1 (w/w) under continuous stirring for 1 h to allow Fe^2+^ adsorption. Subsequently, fresesis steps were conducted under a N_2_ atmosphere to prevent oxidation (Zhang H. et al., 2023; Zhang X. et al., 2023; Zhang M. et al., 2023).

To investigate the effect of composite materials on SRB, as shown in Supplementary Table S3, the materials were added to Starkey’s medium without Fe^2+^ and then added to the sealed culture of SRB bacterial broth. Each treatment was repeated three times at 3 days and 5 days intervals. Samples were collected at 3 days and 5 days for determination of viable bacteria counts using the MPN method and measurement of CAT enzyme activity.

Based on the optimization dosage of the sodium alginate, CaCl_2_, and SRB in the previous research, nZVI@BC was added to the mixture of sodium alginate and SRB at a dosage of 0.1% (w/v). Then, the mixture was dropped into CaCl_2_ to form a composite material immobilized SRB, named SRB@nZVI@BC.

To determine the SO_4_^2−^ reduction capacity of SRB beads and SRB@nZVI@BC, the time when the color of the sample medium turned to different degrees of black was recorded, and the concentration of SO_4_^2−^ in the solution was measured after all the complete culture media had turned dark. The content of SO_4_^2−^ was determined via barium chromate spectrophotometry, with the following steps: 1 g fresh weight SRB beads and SRB@nZVI@BC were placed in 100 mL Starkey medium for sealed culture at 35 °C, and 0.3 mL SRB solution was set as the control treatment; each treatment was repeated three times.

### 2.3 Composite immobilized SRB passivation of heavy metals

#### 2.3.1 Experimental soil

The weakly acidic yellow soil (DK soil) was collected from vegetable fields around a mining area in Dachang Town, Nandan County, Hechi City, Guangxi, and weakly alkaline gray-brown soil (SF soil) was collected from vegetable fields around Diaojiang in Shangfu Village, Du’an County, Hechi City, Guangxi. The physical and chemical properties of the two soils are shown in Supplementary Table S4.

#### 2.3.2 Soil microcosm experiment

We added SRB, SRB beads, and SRB@nZVI@BC to 80 g of contaminated soil (10 mesh) and added 55 mL of liquid, ensuring a 0.5-cm water layer covered the soil surface and submerged the materials. We then sealed the mixture and incubated it at 35 °C. Each treatment was repeated three times. After 7 days of incubation, samples were taken and freeze-dried, and the contents of soil heavy metal species, soil heavy metal leaching toxicity, soil ferrous iron, and soil available sulfur were measured. The treatments in the soil incubation experiment are as follows:

CK: 55 mL sterile H_2_O added to the soil.
Med: 55 mL sterile medium added to the soil.
SRB: 0.6 mL free SRB and 55 mL sterile medium added to the soil.
SS: 2 g SRB beads and 55 mL sterile medium added to the soil.
SSBC-ZVI: 2 g SRB@nZVI@BC and 55 mL sterile medium added to the soil.

#### 2.3.3 Determination of soil properties, soil enzyme activity, and speciation of Cd, Pb, and Sb

Soil pH was measured using a pH meter (S220, Mettler-Toledo, United States), and soil ORP was determined using a pen ORP tester (ORP 100, China). The determination of available sulfur and ferrous iron was conducted as described by Shukla et al. (2021) and de Mello Gabriel et al. (2021). Soil organic matter (SOM) was analyzed using the dichromate oxidation method. We extracted available phosphorus (AP) with 0.5 mol/L HCl and H_2_SO_4_, quantified alkali-hydrolyzable nitrogen (AN) using the alkaline hydrolysis diffusion method, and extracted available potassium (AK) with CH_3_COONH_4_, determining it by flame atomic absorption spectrophotometry (Li et al., 2025). The activities of soil urease (S-UE), soil sucrase (S-SC), and soil catalase (S-CAT) were assessed using specific activity detection kits (Beijing Solarbio Science & Technology Co., Ltd.). Measurements were performed according to the assay protocol with a Tecan Infinite M200 microplate reader (Salzburg, Austria). We fractionated Cd, Pb, and Sb in the soil according to Wang et al. (2021a), separating them into acid-soluble, reducible, oxidizable, and residual fractions. We measured the extracts using ICP-MS.

#### 2.3.4 Related characterization of bio-beads and soil

The structure of SRB@nZVI@BC was analyzed by scanning electron microscopy (SEM, ZEISS Sigma 300). The crystal structure of the soil particles was analyzed using an X-ray diffractometer (XRD, German Bruker D8 Advance), and the functional groups of nZVI@BC, SRB@nZVI@BC, and soil particles were characterized via Fourier transform infrared spectroscopy (FTIR, Thermoscience N10).

#### 2.3.5 Microbial diversity analysis

We sent the freeze-dried soil samples to Majorbio Bio-pharm Technology Co., Ltd.^1^ for 16S rRNA high-throughput sequencing. Microbial DNA was extracted using the E.Z.N.A. OMEGA Soil DNA Kit (Omega Bio-tek, U.S.). The V3–V4 region of the bacterial 16S rRNA gene was amplified using primers 27F (5′-AGAGTT TGATCCTGGCTCAG-3′) and 1492R (5′-TACGGGCTACCTGTT ACGACGACTT-3′). PCR was performed in triplicate. Target bands were purified with the AxyPrep DNA Gel Extraction Kit (Axygen, U.S.) and quantified using a QuantiFluo^™^-ST fluorometer (Promega, U.S.) (Wang et al., 2021b). Taxonomic classification of the 16S rRNA sequences was conducted using the RDP Classifier.^2^

### 2.4 Statistical data analysis

All data related to soil physicochemical properties, heavy metal concentrations and speciation were analyzed using SPSS 26.0. The software MDI Jade 6.5 was used for XRD peak searching, and OMNIC was employed for smoothing, baseline calibration, and peak search in FTIR analysis. The R software was used to analyze microbial sequencing data, and the graphs were generated using Origin 2024b and GraphPad Prism 10.

## 3 Results and discussion

### 3.1 Optimization of preparation parameters for SRB@nZVI@BC

The SEM image of SRB is presented in Supplementary Figure S1, revealing a predominantly curved morphology. The optimal conditions for SRB growth were identified as 10% inoculum, 35 °C, and pH 7 (Supplementary Figure S2). All subsequent experiments were conducted accordingly. The preparation conditions have a significant influence on the performance of immobilized SRB beads. In this study, nine types of immobilized SRB beads (1–9) were prepared using the L9 (3^4^) orthogonal design; their morphology is shown in Supplementary Figure S3A, and their physical properties are shown in Supplementary Text S2. Supplementary Table S5 shows that the beads’ pore sizes are mainly approximately 2–50 nm, indicating a mesopore-predominant gel material (Karunaratne et al., 2022). The adsorption–desorption curves are consistent with type IV in the IUPAC classification (Peng et al., 2024). Supplementary Table S6 indicates that a larger specific surface area is more conducive to mass transfer performance. In the mass transfer performance test of nine SRB beads, the concentration of SA significantly affected the mass transfer performance of the beads. According to Supplementary Table S7, the factors affecting mechanical strength were ranked in order of influence as follows: SA concentration > CaCl_2_ concentration > SRB bacterial solution concentration. Sodium alginate (SA) forms a gel matrix through ionic crosslinking with Ca^2+^, effectively encapsulating the SRB. The concentrations of SA and CaCl_2_ determine the gel’s stability and mechanical properties, significantly impacting SRB activity (Sun et al., 1989). Chang and Chou (2002) reported that high SA concentrations increase particle size and strength but impede SO_4_^2−^ diffusion and H_2_S release, leading to reduced water content and immobilized bacterial death. At 120 h, the SO_4_^2−^ reduction rates for the nine beads were 45.14, 49.19, 42.06, 40.46, 42.89, 33.91, 35.06, 34.50, and 31.23%, respectively (Supplementary Table S8). Among these, the formulation #2 beads exhibited the best performance, achieving a reduction rate of 49.19%, which was 24.34% higher than that of free SRB (Supplementary Figure S3C).

In this study, combining mass transfer performance test, mechanical strength, and the SO_4_^2−^ reduction rates, the optimal preparation conditions for the immobilized SRB beads were 2% SA, 2% CaCl_2_, and 30% SRB inoculum concentration. These conditions offered a balance between mechanical strength, particle size, and mass transfer capacity, thereby enhancing sulfate reduction efficiency (Zhao C. et al., 2021; Zhao Q. et al., 2021).

**Figure 1:**
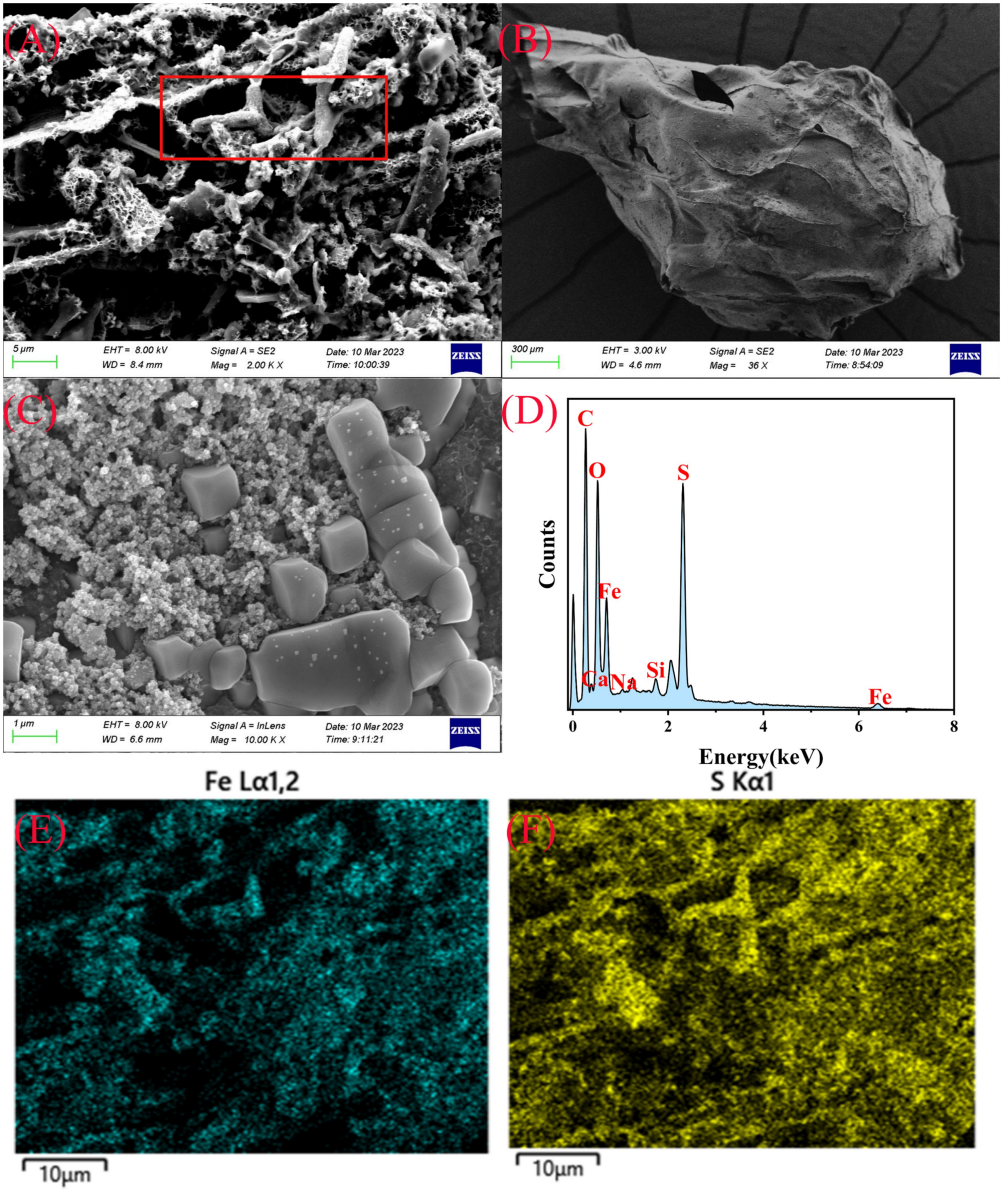
Scanning electron microscope photos of SRB@nZVI@BC **(A–C)**. EDS of SRB@nZVI@BC **(D)**. Element distribution for S element **(E)** and Fe element **(F)**.

Based on the optimized preparation conditions for immobilized SRB beads, composite immobilized SRB beads were prepared by adding nZVI@BC and termed SRB@nZVI@BC (Supplementary Figure S3D). Analysis of catalase (CAT) enzyme activity, and MPN (most probable number) of viable bacteria (Supplementary Text S3) showed that at a composite material concentration of 0.1% (w/v), SRB growth was promoted, with a more favorable effect than at a 0.5% concentration. The BC is alkaline and provides suitable adsorption sites for bacteria, thereby facilitating bacterial growth (Figures 1A–D). When an appropriate amount of BC is added, a favorable acidic and alkaline buffer environment for SRB is created, enhancing their density (Lu et al., 2020). The nZVI@BC material contains zero-valent iron (Figures 1D–F), which reduces and consumes oxygen during oxidation, lowering the oxidation–reduction potential in the liquid environment and, thus, stimulating SRB growth. Therefore, the composite material at a concentration of 0.1% promotes SRB growth (Zhao C. et al., 2021; Zhao Q. et al., 2021).

In this study, SRB@nZVI@BC’s sulfate reduction rate was higher than that of SRB beads and SRB load with biochar (SSBC) (Supplementary Figure S5), indicating that an appropriate amount of the composite material can stimulate SRB growth and dissimilatory reduction. The time required to generate FeS black precipitate further validated these results.

### 3.2 Sulfide mineralization and soil functional restoration by SRB@nZVI@BC

#### 3.2.1 The impact of SRB@nZVI@BC on soil properties and enzyme activity

**Figure 2:**
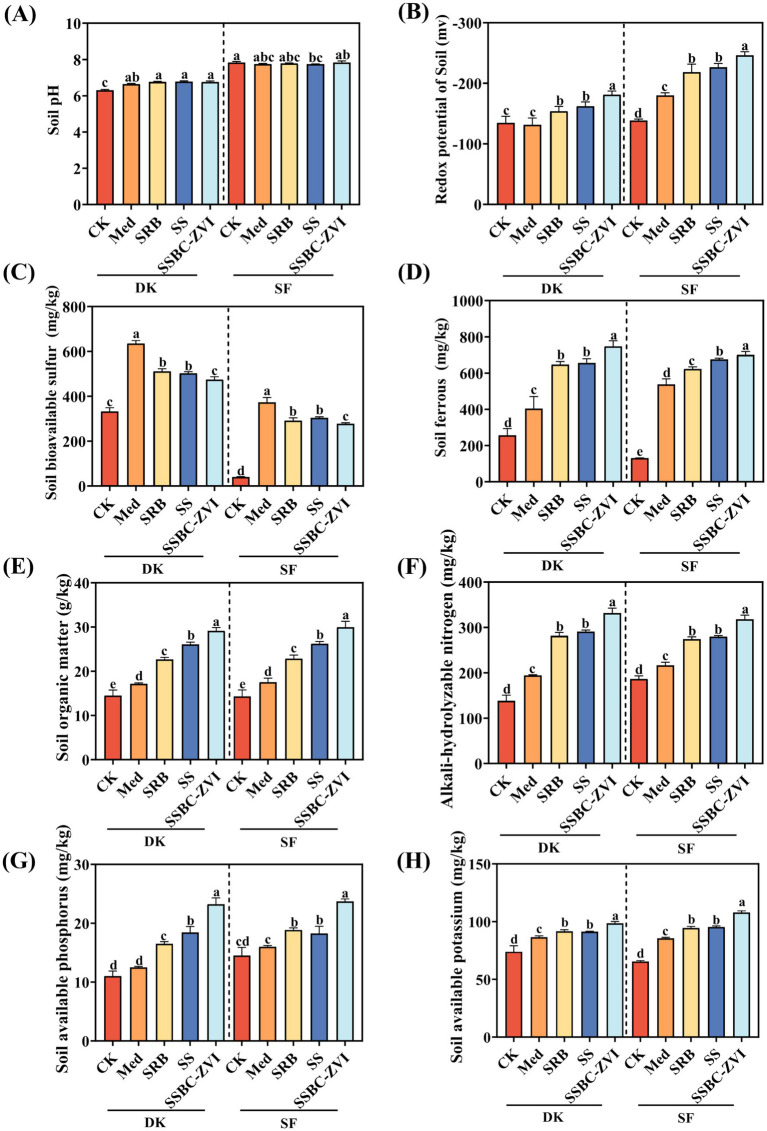
Effects of different treatments on soil pH **(A)**, soil redox potential **(B)**, soil bioavailable sulfur **(C)**, soil ferrous **(D)**, soil organic matter **(E)**, alkali-hydrolyzable nitrogen **(F)**, soil available phosphorus **(G)**, and soil available potassium **(H)**.

**Figure 3:**
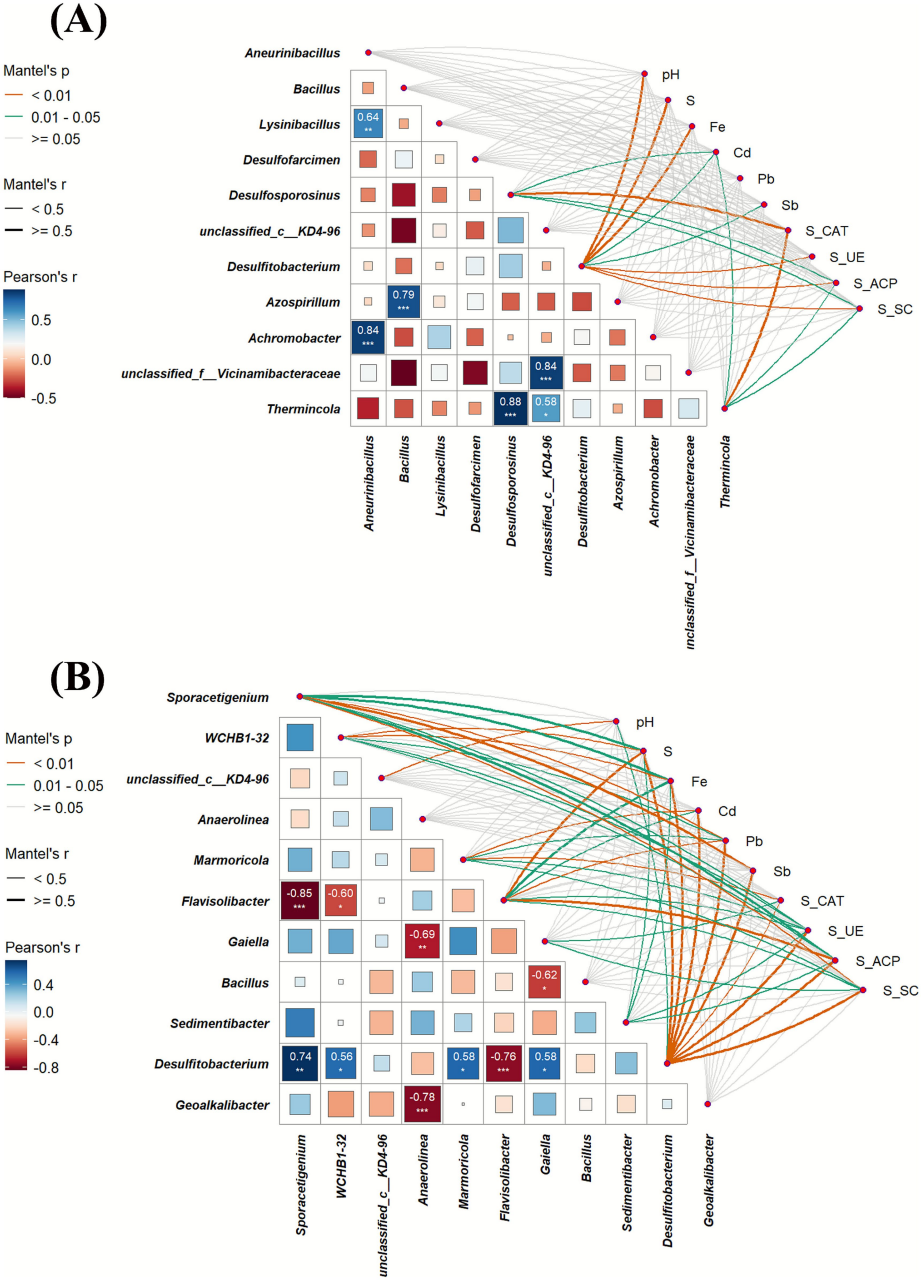
Mantel correlation analysis in DK **(A)** soil and SF **(B)** soil. Relationship between environmental variables (soil properties) and top 10 soil bacteria, generated by the Mantel test. Pairwise comparisons of the environmental variables are displayed with a color gradient denoting Spearman’s correlation coefficient. The level of correlation significance is indicated by asterisks: ^***^for *p* < 0.001 and ^*^for *p* < 0.05.

Heavy metal pollution not only exerts significant toxic effects on plants and animals, but also disrupts the ecological function of soils by altering their physicochemical properties (e.g., pH and organic matter), thereby inhibiting plant growth and development (Si et al., 2023). Such effects are particularly pronounced under conditions of combined pollution with multiple heavy metals. Therefore, when conducting soil remediation for heavy metal pollution, it is crucial to simultaneously focus on improving soil physicochemical properties to enhance its ecological function and productivity. In this study, SRB, SS, and SRB@nZVI@BC were used to remediate soil contaminated with Cd, Pb, and Sb. Increasing soil pH correlated with decreased soil redox potential (ORP), increased ferrous iron content, and decreased bioavailable sulfur content (Figures 2A–D). Compared to the control (CK), the application of SRB@nZVI@BC significantly improved soil fertility. Organic matter content increased by 60–65%, while alkali-hydrolyzable nitrogen, available phosphorus, and available potassium increased by 133–140%, 103–110%, and 125–210%, respectively (Figures 2E–H). Analysis of soil enzyme activity showed that amendment application significantly enhanced enzymatic activities (Supplementary Figure S6). Compared to CK, SRB@nZVI@BC increased catalase, urease, phosphatase, and sucrase activities by 7.85–9.47%, 169.88–176.20%, 107.62–199.17%, and 134.18–202.28%, respectively. In this study, the application of SRB@nZVI@BC significantly improved soil-related physicochemical indicators, such as SOM, AN, AK, and AP (Figure 2). Biochar, as a widely applied soil amendment, is inherently rich in phosphorus and potassium. When incorporated into soils, it not only significantly improves physicochemical properties—such as enhancing SOM, total phosphorus (TP), and total potassium (TK) levels—but also further increases the overall nutrient reserves of the soil (Li et al., 2023; Parasar and Agarwala, 2025). In addition, the application of biochar promotes an increase in soil pH, which notably decreases the bioavailability of soil heavy metals (Qi et al., 2021). The incorporation of SRB@nZVI@BC significantly enhanced the activities of several key soil enzymes, including sucrase, urease, and phosphatase (Supplementary Figure S6). Elevated enzyme activities in SRB@nZVI@BC-treated soils indicate stimulated microbial metabolism and enhanced nutrient (C, N, and P) cycling. These enzymes, widely regarded as indicators of soil health, were positively correlated with SRB abundance and critical soil chemical parameters (Figure 3). This suggests that SRB-driven sulfur reduction creates microenvironments favorable for enzymatic processes, while nZVI@BC provides both electron donors and habitat support for microbial communities (Dai et al., 2021). Moreover, in the core metabolic process of SRB, SO_4_^2−^ is reduced to S^2−^, a strong reductant that can convert insoluble FePO_4_ in soils into soluble Fe_3_(PO_4_)_2_ and combine with Fe^2+^ to form FeS precipitates (Supplementary Figure S9), thereby increasing the content of available phosphorus. At the same time, the organic acids released during SRB metabolism can chelate metal cations that immobilize phosphorus and potassium, thereby liberating the bound nutrients and significantly enhancing the levels of available phosphorus and exchangeable potassium in soils. Therefore, SRB@nZVI@BC improves soil physicochemical properties, enhances enzyme activity, and promotes nutrient availability, resulting in a comprehensive improvement in soil fertility. Meanwhile, by reacting with heavy metal ions to form stable precipitates, it reduces the bioavailability of heavy metals, reinforcing its dual role in contaminant remediation and soil ecological restoration. Collectively, these mechanisms demonstrate that SRB@nZVI@BC holds great potential as a sustainable and efficient strategy for the remediation of heavy metal-contaminated soils.

#### 3.2.2 Effect of SRB on immobilization of heavy metals in soil

**Figure 4:**
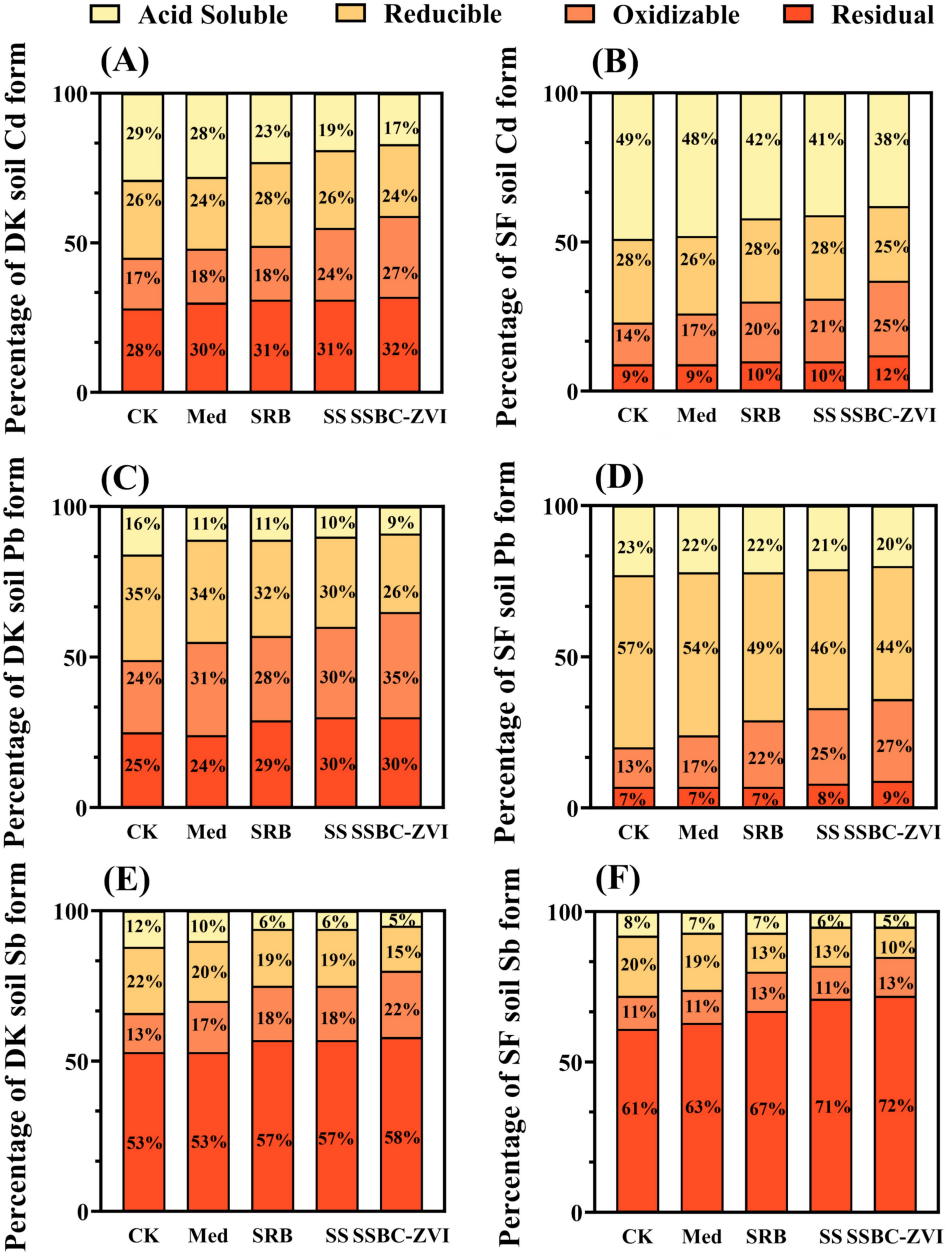
Speciation distribution of Cd **(A,B)**, Pb **(C,D)**, and Sb **(E,F)** in soil in DK and SF treatments.

Removal efficiency from soil water leachate by SRB@nZVI@BC ranged from 60.22 to 63.93% for Cd, 57.13 to 59.45% for Pb, and 56.02 to 70.37% for Sb (Supplementary Figure S7). The percentages of Cd, Pb, and Sb in their stabilized forms (residual and oxidizable) increased with SRB addition. Under SRB@nZVI@BC treatment, the percentage of exchangeable Cd (acid-soluble and reducible) decreased by 14% in both DK and SF soils. Similarly, exchangeable Pb content decreased by 16% in both soils, while exchangeable Sb content decreased by 14 and 13% in DK and SF soils, respectively (Figure 4). The treatment effectively reduced the mobility and toxicity of Cd, Pb, and Sb. After SRB@nZVI@BC treatment, Cd, Pb, and Sb mobility and transformation were significantly reduced.

Microorganisms can remove heavy metals from the environment through various mechanisms, including biomineralization, bio-reduction, adsorption, and bioaccumulation (Song et al., 2022). Supplementary Figure S8 shows the XRD diffraction patterns of soil samples before and after treatment with DK and SF. We observed strong diffraction peaks at 2θ = 36.46°, 39.44°, and 59.90°. These peaks correspond to high matches with PDF card results for CdS, Pb_4_Sb_6_S_13_, and PbS, respectively (Chang et al., 2025; Cheng et al., 2023; Zang et al., 2023). The presence of CdS, Pb_4_Sb_6_S_13_, and PbS indicates the formation of stable minerals involving Cd, Pb, and Sb under bacterial activity. The presence of CdS, Pb_4_Sb_6_S_13_, and PbS confirms that SRB use SO_4_^2−^ as an electron acceptor, reducing sulfate to S^2−^ when lactate is used as a substrate. This S^2−^ then reacts with Cd, Pb, and Sb in the soil to form CdS, PbS, and Sb_2_S_3_. These findings are consistent with the hypothesis that SRB play a key role in transforming heavy metals into less mobile and less toxic forms, which is crucial for soil remediation.

**Figure 5:**
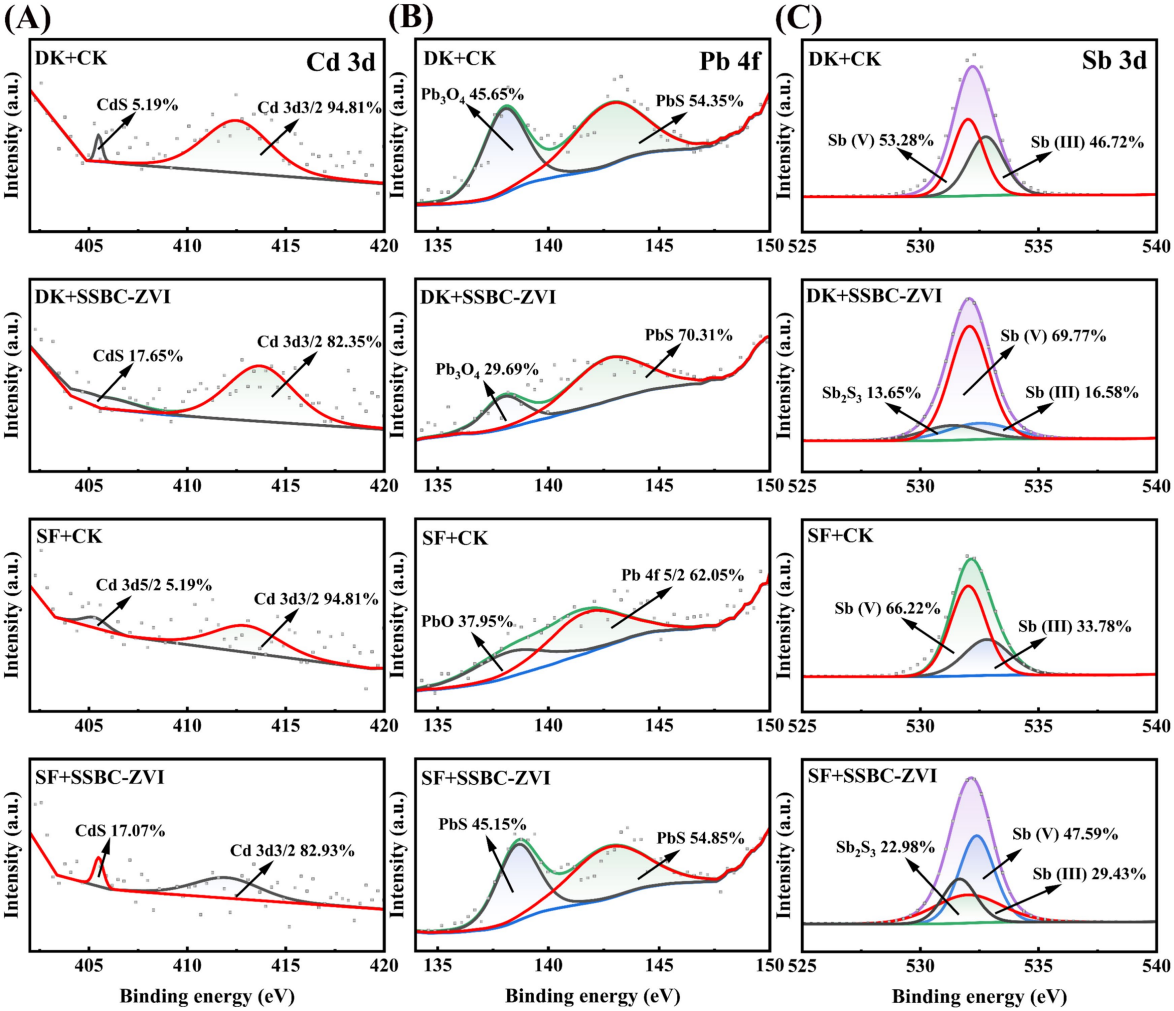
**(A–C)** XPS spectra of soils after treatment with different remediation materials **(A–C)**: **(A)** Cd 3d, **(B)** Pb 4f, and **(C)** Sb 3d.

The XPS analyses further confirmed this conclusion (Figure 5). In the S2p peak, adding SRB@nZVI@BC increased the total percentage of CdS, PbS, and Sb_2_S_3_ by 7.37% in DK soil and 10.95% in SF soil (Supplementary Figure S9A). In the Fe2p peaks, SRB@nZVI@BC addition significantly increased FeS peak occupancy by 1.91% in DK soil and 2.77% in SF soil (Supplementary Figure S9B). Within the Cd3d, Pb4f, and Sb3d peaks, SRB@nZVI@BC addition increased CdS, PbS, and Sb_2_S_3_ peaks by 12.46, 15.96, and 13.65% in DK soil, respectively, and by 17.07, 54.85, and 22.98% in SF soil, respectively. These substantial increases in the intensities of metal sulfide peaks further indicate the effective reduction and precipitation of toxic metals in the soil under SRB and nZVI influence, which is key for effective heavy metal immobilization.

#### 3.2.3 The synergistic mechanism of SRB@nZVI@BC

In practical applications, the remediation effect of SRB is not always significant, which may be due to environmental conditions that are unsuitable for SRB growth, such as heavy metal stress, too low or too high pH values, ORP, and unsuitable electron donors (Yang et al., 2024). In this study, the application of SRB@nZVI@BC significantly altered the soil pH, ORP, bioavailable sulfur, and Fe^2+^ concentrations (Figure 2). These changes are primarily attributed to the function of nZVI, which regulates the soil redox environment and supports SRB activity. nZVI has strong reducing properties and consumes dissolved oxygen, thereby lowering ORP and creating anaerobic conditions that facilitate SRB growth (Dong et al., 2019). Additionally, the H_2_ produced during the oxidation of ZVI can serve as an electron donor for SRB, enhancing the competitive advantage of SRB (Liang et al., 2021). Moreover, the SRB@nZVI@BC treatment led to significantly elevated Fe^2+^ concentrations compared to other treatments. This indicates continuous ZVI oxidation and the generation of reducing equivalents. These nZVI-induced chemical changes create a favorable niche for SRB growth and enhance their dissimilatory sulfate reduction. As a result, the immobilization of heavy metals in soil is effectively promoted through these synergistic chemical–microbial interactions.

As shown in Figure 1B, the biochar possesses a rough and porous surface morphology, which not only provides abundant sites for the attachment of SRB but also creates favorable microenvironments that support their colonization and proliferation within the pore structure. Such a highly porous architecture enhances the physical entrapment and spatial distribution of SRB, thereby strengthening the stability of the microbial–material interface. Moreover, the encapsulation with SA serves as an additional protective barrier, effectively mitigating the direct toxic effects of heavy metals on SRB cells. This dual protection, arising from both the structural characteristics of biochar and the shielding capacity of alginate, contributes to the sustained viability and metabolic activity of SRB, which is crucial for maintaining efficient heavy metal immobilization and removal. When nZVI@BC is added to immobilized SRB beads, the loose, porous surface and dense internal channels of biochar provide growth sites for SRB, whereas the biochar structure and its abundant functional groups facilitates the adsorption of heavy metals at specific sites, reducing the direct contact between SRB and heavy metal ions.

To investigate the role of functional groups in the remediation of Cd, Pb, and Sb, characterization was performed on both materials and soils. Results showed that nZVI@BC and SRB@nZVI@BC were rich in oxygen-containing functional groups, such as C–O–C, O=C–O and Fe–O (Supplementary Figure S10). The addition of SRB and SA increased the abundance of these groups. In soil, SRB@nZVI@BC significantly increased the content of oxygen-containing functional groups. SRB generate volatile fatty acids and other organic acids during metabolism, which can transiently regulate the solution pH and induce protonation/deprotonation of functional groups on biochar surfaces, thereby altering their surface charge and reactivity (Zhang et al., 2022). In addition, acid-mediated surface oxidation or acid modification increases oxygen-containing functional groups such as carboxyl and phenolic hydroxyl groups (Supplementary Figure S10), which typically confer a higher cation exchange capacity (CEC) under neutral conditions (Wu et al., 2019). Consequently, this enhances the adsorption of heavy metal ions onto biochar. Moreover, the coupling of SRB with biochar provides a favorable microenvironment and electron transfer pathways, while synergizing with sulfide precipitation processes, ultimately improving the overall efficiency of heavy metal removal. The addition of SRB@nZVI@BC increased the C–O–C and O=C–O peaks from 13.35 to 16.52% and 8.57 to 10.04% in DK soil, and from 10.75 to 18.26% and 8.06 to 10.84% in SF soil, respectively (Supplementary Figure S9). These oxygen-containing functional groups enhance the availability of electron donors, which are crucial for SRB to reduce SO_4_^2−^ to S^2−^. The generated S^2−^ then reacts with metal ions in the soil to form stable metal sulfides, such as CdS, PbS, and Sb_2_S_3_ (Supplementary Figure S9) (Yuan et al., 2018). In addition, these functional groups provide active sites for the adsorption of heavy metals, further facilitating metal immobilization. Through mechanisms such as coordination and ionic exchange, these groups likely play a key role in stabilizing metal ions before their reduction to less toxic forms (Guan et al., 2018; Munir et al., 2020).

nZVI promotes SRB growth and enhances their tolerance to metal toxicity. Meanwhile, the porous structure and abundant surface functional groups of biochar provide a favorable microenvironment for SRB attachment and proliferation, further improving metal removal efficiency. During metabolism, SRB produce organic acids that regulate pH and facilitate the protonation/deprotonation of surface groups. In addition, SRB-mediated oxidation of biochar’s organic components increases the abundance of oxygen-containing functional groups, thereby enhancing its adsorption capacity. The synergistic interactions among nZVI, SRB, and biochar collectively contribute to the superior and stable immobilization of heavy metals by the SRB@nZVI@BC composite in contaminated soils. This synergistic mechanism highlights the distinct advantage of the SRB@nZVI@BC system over conventional single-component remediation strategies. Meanwhile, the composite significantly improved key soil fertility indicators (organic matter, alkali-hydrolyzable nitrogen, available phosphorus, and available potassium) (Figures 2A–D), demonstrating its dual role in soil remediation and fertility enhancement.

### 3.3 Soil microbial community assembly patterns driven by SRB@nZVI@BC

**Figure 6:**
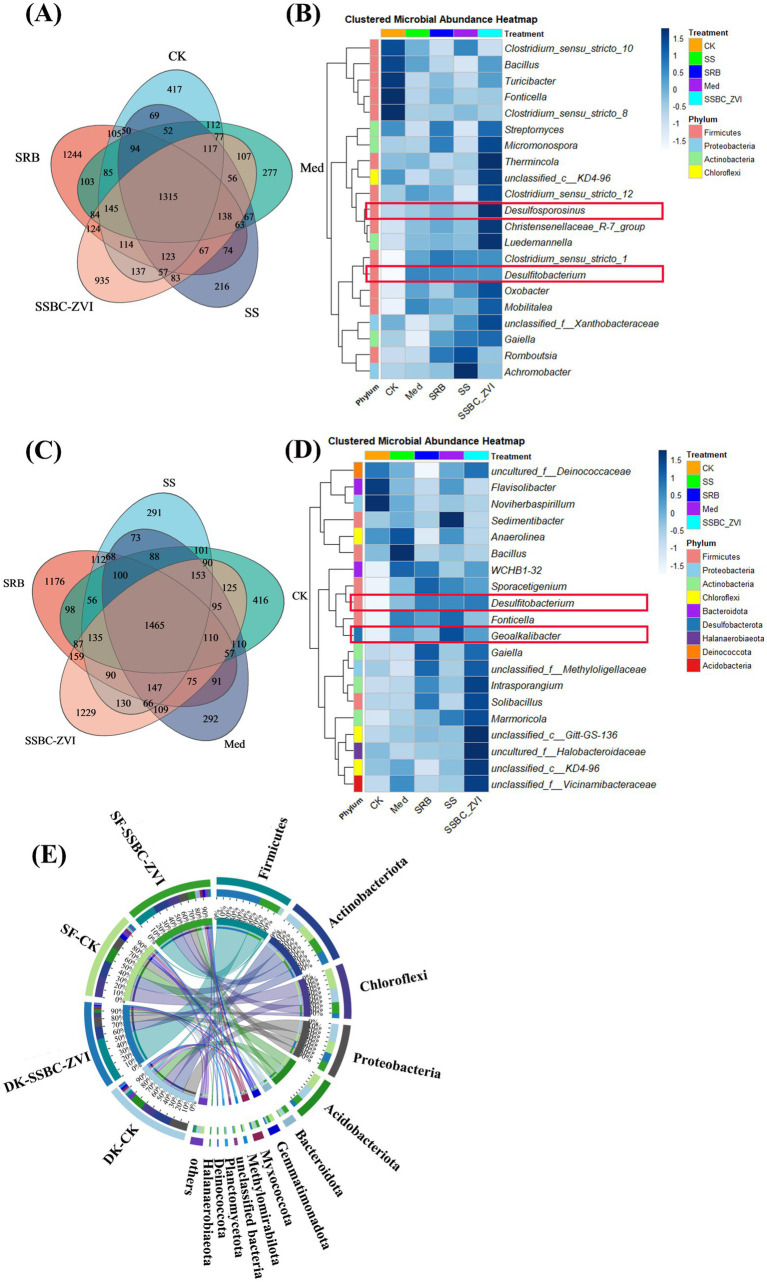
Venn plots of microbial diversity under CK, Med, SRB, SS, and SSBC-ZVI treatments in **(A)** DK soil and **(C)** in SF soil; composition changes in the top 20 bacteria at the genus level in **(B)** DK soil and **(D)** in SF soil. **(E)** Composition changes in the top 10 and other soil bacteria at the phylum level in DK soil and SF soil.

The effects of different treatments on soil microbial communities were evaluated using 16S rRNA gene sequencing. Distinct shifts in microbial composition were observed in both DK and SF soils (Figure 6).

At the phylum level, the dominant taxa in both DK and SF soils included Firmicutes, Proteobacteria, Actinobacteria, and Chloroflexi, together accounting for 87.06 to 91.26% of the total relative abundance.

In DK soil, the top 10 genera included *Clostridium_sensu_stricto_10*, *Bacillus*, *Oxobacter*, *Thermincola*, *Desulfofarcimen*, and *Desulfitobacterium*, among others. Notably, *Desulfofarcimen*—a key sulfate-reducing bacterium (SRB)—showed significantly higher abundance in the SRB@nZVI@BC treatment (14.57%), indicating stimulated SRB proliferation (Figure 6C). The top 10 genera in SF soil included *Bacillus*, *Sporacetigenium*, *Flavisolibacter*, *Anaerobia*, and *norank_f_norank_0_Subgroup_7*, among others, accounting for 45.64 to 51.79% of the total relative abundance. *Desulfosporosinus*, which is associated with sulfate reduction, ranked 22nd in relative abundance, with the highest relative abundance observed in the SRB@nZVI@BC treatment (Figure 6E).

Functional annotation using FAPROTAX identified four sulfur cycle-related pathways: respiration of sulfur compounds, sulfate respiration, sulfur respiration, and thiosulfate respiration. These functions were significantly enhanced in both soils following SRB@nZVI@BC application.

Mantel correlation analysis revealed positive associations between soil pH, ferrous iron content, enzyme activities, and the abundance of *Desulfitobacterium* and *Desulfosporosinus*. Additionally, these genera were positively correlated with the oxidized forms of Cd, Pb, and Sb.

**Figure 7:**
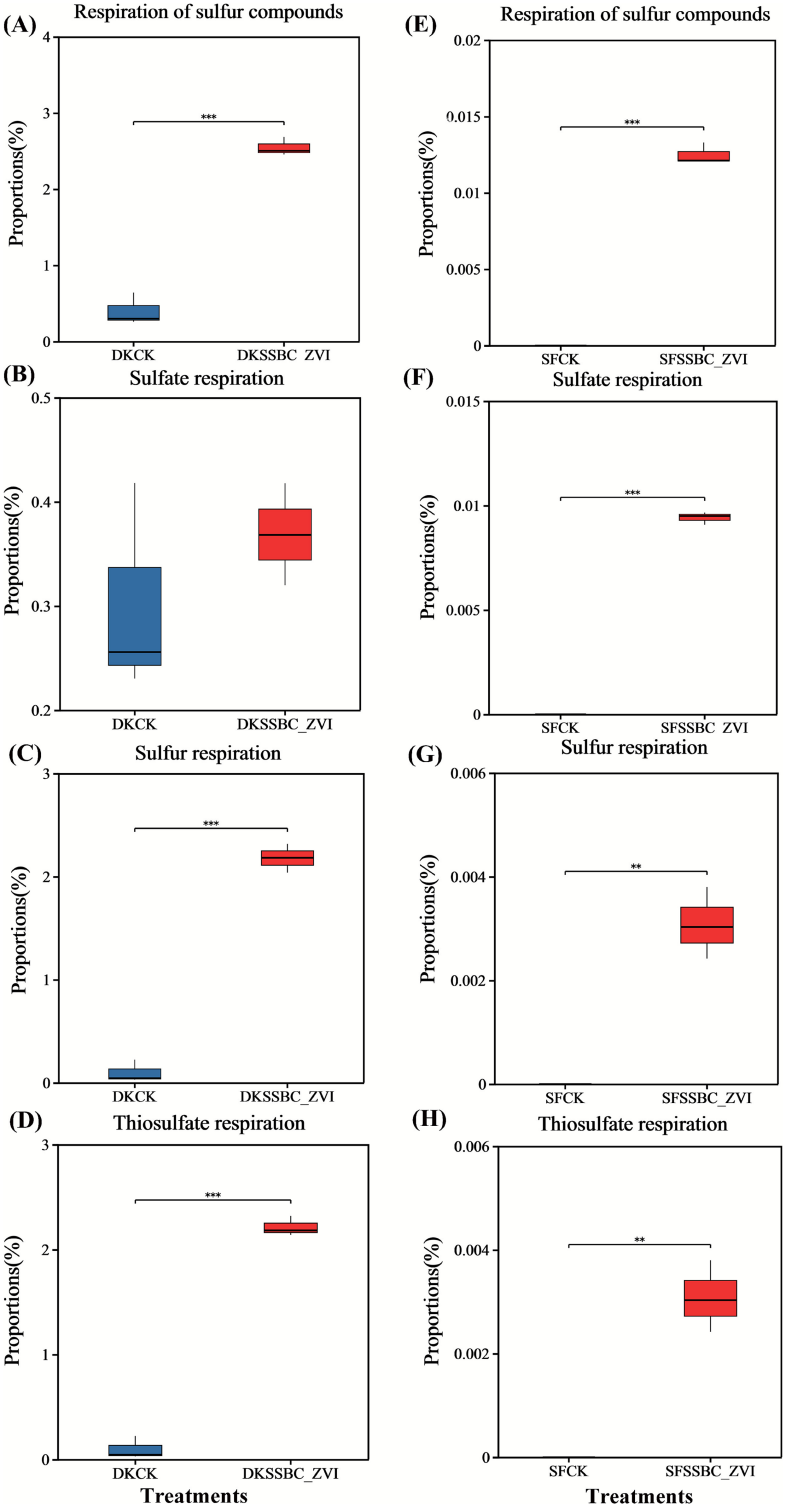
FAPROTAX functional annotation analysis of four sulfur cycle-related functions: **(A–D)** in DK soil and **(E–H)** in SF soil. The level of correlation significance is indicated by asterisks: ^***^for *p* < 0.001 and ^*^for *p* < 0.01.

Soil microbial communities regulate the cycling and availability of soil organic matter and essential nutrients, the biodegradation of harmful organic pollutants, and the biotransformation of toxic metals, thereby serving as a primary driver of long-term sustainability in terrestrial ecosystems (Azadi and Raiesi, 2021). The application of SRB@nZVI@BC significantly increased the ace, Chao1, and Shannon indices (Supplementary Table S10), indicating an enhancement of rhizosphere microbial diversity. Improved soil microbial diversity, in turn, facilitates C, N, and P cycling as well as enzymatic activities, ultimately contributing to higher soil fertility. In the SRB@nZVI@BC treatments, phyla such as Firmicutes, Bacteroidetes, and Proteobacteria were dominant (Figure 6E). This trend aligns with previous findings showing Firmicutes, Bacteroidetes, and Proteobacteria as dominant taxa in heavy metal-contaminated soils (Liu et al., 2024; Liu et al., 2020). Firmicutes, in particular, are classified as r-strategists and primarily found in favorable environmental conditions (Jin et al., 2023). At the genus level, the addition SRB@nZVI@BC significantly increased the relative abundances of *Desulfosporosinus*, *Desulfitobacterium* (belonging to Firmicutes), and *Geoalkalibacter* (belonging to Desulfobacterota). The increase in sulfate-reducing bacterial genera promotes the reduction of SO_4_^2−^ to HS^−^ or S^2−^, which reacts with heavy metal ions such as Cd, Pb, and Sb to form stable sulfide precipitates. FAPROTAX analysis confirmed this view (Figure 7). Sulfate respiration is one of the most critical metabolic pathways of SRB. SRB can reduce SO_4_^2−^ to HS^−^ or S_2_^2−^, and these reduced products readily react with heavy metal ions such as Cd^2+^, Pb^2+^, and Sb^3+^ to form stable metal sulfide precipitates, thereby significantly decreasing the bioavailability and mobility of heavy metals (Zhang H. et al., 2023; Zhang X. et al., 2023; Zhang M. et al., 2023). At the same time, the activation of the sulfite respiration pathway also plays an important role. SO_3_^2−^ and its intermediate, S_2_O_3_^2−^, often exist in soils as transitional states, and their further reduction generates HS^−^, serving as an important supplementary source driving heavy metal sulfide precipitation. FAPROTAX analysis indicated that the application of the SRB@nZVI@BC composite significantly enhanced the metabolic activity of SRB, thereby accelerating both sulfate and sulfite reduction processes and amplifying the reactions between heavy metals and sulfur reduction products. Consequently, Cd^2+^, Pb^2+^, and Sb^3+^ ions were transformed into more stable sulfide precipitates, which represents the key mechanism by which the composite material exerts its core role in the immobilization of heavy metals in soil. Besides, Mantel analysis further revealed the correlations between SRB and environmental factors as well as heavy metal speciation. Among them, key genera such as *Desulfosporosinus* and *Desulfitobacterium* showed significant positive correlations with soil pH, sulfur (S), iron (Fe), and the oxidizable states of Cd, Pb, and Sb (Figure 3). This indicates that the application of the composite not only promoted the enrichment of sulfate-reducing bacteria but also enhanced their metabolic activity by altering soil physicochemical properties (e.g., increasing pH and the availability of sulfur and iron). Elevated soil pH and sulfur supply provide favorable conditions for the growth and respiratory metabolism of SRB, while the presence of iron may facilitate electron transfer and metal reduction processes. Meanwhile, the positive correlations between the oxidizable states of Cd, Pb, and Sb and SRB abundance suggest that SRB activity is closely linked to the transformation of these heavy metals, likely through promoting sulfide precipitation and interacting with iron, thereby contributing to heavy metal stabilization. This finding is consistent with the FAPROTAX analysis, further demonstrating that the SRB@nZVI@BC composite can enhance the functional capacity of sulfate-reducing bacterial communities and improve soil environmental conditions, thereby achieving efficient heavy metal immobilization through multiple pathways. Genera such as *Marmoricola*, *Sedimentibacter*, and *Sporacetigenium* were positively associated with both the oxidized forms of Cd, Pb, and Sb and with the presence of *Desulfitobacterium* (Figure 3), suggesting that the addition of SRB also activates other microorganisms associated with the oxidation of Cd, Pb, and Sb, contributing to the co-passivation of heavy metal ions. This finding aligns with the results of Li et al. (2021) and Pan et al. (2020). Genera such as *Sedimentibacter* are associated with the reduction of Fe(III), and sulfate-reducing bacteria frequently reduce both sulfate and Fe(III) simultaneously. In this reduction process, Fe can interact with Cd, Pb, and Sb ions to form more stable iron mineral phases, promoting the fixation of soil heavy metals (Lv et al., 2021; Xue et al., 2018). Soil XRD analysis further corroborated this conclusion.

The SEM structural equation model (Supplementary Figure S11) revealed that the levels of oxidized Cd, Pb, and Sb in the soil were significantly influenced by the soil ferrous iron content, the bioavailable sulfur level, and the abundance of SRB. Among these, the effect of ferrous iron was most pronounced. Bioavailable sulfur in the soil was negatively correlated with the oxidized forms of Cd, Pb, and Sb. Both ferrous iron and bioavailable sulfur significantly influenced SRB growth, which is consistent with our experimental results. The application of SRB@nZVI@BC significantly increased the ferrous iron content and reduced the bioavailable sulfur content in the soil. The addition of nZVI further promoted SRB growth (0.32).

In summary, SRB@nZVI@BC outperformed SRB alone in remediating co-contaminated soils by promoting beneficial microbial communities, improving soil biochemical functions, and enhancing the cycling of C, N, and P. However, this study was conducted under controlled laboratory conditions, and thus the results may not fully represent the complexity of field environments. The potential toxicity associated with nZVI input warrants caution. Future work should aim to optimize the nZVI dosage, evaluate long-term environmental impacts, and assess the economic viability of large-scale applications to ensure safe and sustainable use.

## 4 Conclusion

In this study, SRB@nZVI@BC were developed by immobilizing SRB onto SA and nZVI@BC via microbial immobilization technology. The optimal formulation consisted of 2% SA, 2% CaCl_2_, 30% SRB cell suspension, and 0.1% nZVI@BC. The synthesized SRB@nZVI@BC demonstrated effective remediation of Cd-, Pb-, and Sb-contaminated soils. Mechanistic investigations revealed that the addition of nZVI@BC enhanced SRB activity, promoting sulfur cycling and the generation of S^2−^, which facilitated heavy metal precipitation as insoluble sulfides. Additionally, SRB@nZVI@BC enhances the soil’s heavy metal adsorption capacity by activating oxygen-containing functional groups, such as C-O-C. SRB@nZVI@BC reshaped the soil microbial community by enriching sulfate-reducing genera such as *Desulfosporosinus* and *Desulfitobacterium*, which played a key role in driving heavy metal transformation and stabilization. The composite also improved the availability of soil nutrients (N, P, K) and increased enzyme activities, contributing to soil fertility recovery. Overall, by coupling chemical stabilization with biologically driven ecological restoration, SRB@nZVI@BC offers a promising strategy for simultaneous heavy metal immobilization and improvement of soil health under multi-metal contamination stress.

## Data Availability

The data analyzed in this study is subject to the following licenses/restrictions: data will be made available on request. Requests to access these datasets should be directed to 18277839022@163.com.

## Footnotes

1
http://www.majorbio.com

2
http://rdp.cme.msu.edu/index.jsp
