# Supplementary material for: Enhanced immobilization of cadmium, lead, and antimony with improved soil fertility using sulfate-reducing bacteria@nano zero-valent iron-modified biochar: coupled chemisorption and microbial mechanisms
Source: Front Microbiol. 2026 Jan 5;16:1712696. doi: 10.3389/fmicb.2025.1712696 (PMC12812550; doi:10.3389/fmicb.2025.1712696)
Supplement: Supplementary file 1 [file Data_Sheet_1.docx]

**Supplementary Information for**

Enhanced immobilization of cadmium, lead, and antimony with improved soil fertility using sulfate-reducing bacteria@nano zero-valent iron-modified biochar: Coupled chemisorption and microbial mechanisms

**Authors:**

Shirui Peng^a†^, Fengshuo Ya^a†^, Juan Yin^b^, Changjun Liao^b^, Dangling Qin^b^, Jiapan Lian^a^, Hong Li^c^, Hailong Wang^d^, Jianming Xue^e^, Xiaoe Yang^f^, Hongfei Lin^b^, Jiancheng Chen^a^, Guofei Pan^a^, Yanyan Wei ^a*^

**Author Affiliations:**

^a^ State Key Laboratory for Conservation and Utilization of Subtropical Agri–bioresources, Guangxi Key Laboratory for Agro-Environment and Agro-Products Safety, National Demonstration Center for Experimental Plant Science Education, College of Agriculture, Guangxi University, Nanning 530004, China.

^b^ Bossco Environmental Protection Technology Co., Ltd., Nanning 530004, China.

^c^ Key Laboratory of Eco-Environment of Three Gorges Region, Ministry of Education, Chongqing University, Chongqing 400044, China

^d^ School of Environment and Chemical Engineering, Foshan University, Foshan, 528000, China.

^e^ New Zealand Forest Research Institute (Scion), Forest System, POB 29237, Christchurch 8440, New Zealand.

^f^ Ministry of Education Key Laboratory of Environmental Remediation and Ecological Health, College of Environmental and Resource Sciences, Zhejiang University, Hangzhou 310058, China.

† Equal contribution and first authorship

**^*^Correspondence:**

Dr. Yanyan Wei

College of Agriculture, Guangxi University, Nanning 530004, P. R. China

Tel: +86–18607718450;

E–mail: yanyanwei@gxu.edu.cn

**Contents**

**Text**

**Text S1** Evaluation of mechanical and mass transfer properties

**Text S2** Determination of mechanical properties of immobilized SRB beads

**Text S3** Effect of composites on SRB catalase activity and viable cell count (MPN)

**Table**

**Table S1** Composition of improved Starkey medium

**Table S2** Orthogonal design table

**Table S3** Effect of composite materials on the growth of SRB

**Table S4** Physicochemical properties of tested soils

**Table S5** Analysis of specific surface area of immobilized SRB beads

**Table S6** Intuitive analysis table of orthogonal design with mass transfer performance as index

**Table S7** Orthogonal design intuitive analysis table of ball with mechanical strength as index

**Table S8** Visual analysis table of orthogonal design of small balls with SO_4_^2-^ reduction rate

**Table S9** Effect of composites on SRB catalase activity and viable cell count (MPN)

**Figure**

**Fig. S1** SEM image of SRB.

**Fig. S2** The changes of SRB at different inoculation amount (A), initial pH (B), and temperatures (C).

**Fig. S3** Morphology of immobilized SRB beads (A), adsorption-desorption isotherms of different immobilized SRB beads (B), formation of FeS under the cultivation of immobilized SRB beads (C) and morphology of immobilized SRB beads (D).

**Fig. S4** Determination of mass transfer properties of immobilized SRB spheres.

**Fig. S5** Production of FeS by three immobilized SRB beads.

**Fig. S6** Effect of different treatments on the activities of catalase (A), urease (B), acid phosphatase (C) and sucrase (D)in DK and SF soils.

**Fig. S7** Effects of different treatments on Cd (A), Pb (B), and Sb (C) concentrations in soil water leachate.

**Fig. S8** FITR (A), XRD (B) and XPS (C) in diffraction pattern analysis of soil particles.

**Fig. S9** (A)-(C) are XPS spectra of different adsorbent materials: (A) C1 s, (B) S 2p, and (C) Fe 2p.

**Fig. S10** FITR of nZVI@BC and SRB@nZVI@BC.

**Fig. S11** Structural Equation Model illustrating the effects of soil properties (environment) and SRB on the oxidation of Cd, Pb, and Sb. The paths with insignificant coefficients are not displayed; orange and blue arrows indicate positive correlations, and dashed lines represent negative correlations.

**Text S1** Evaluation of mechanical and mass transfer properties

For mechanical strength, a single bead was placed at the junction of a 10-mL syringe barrel and needle; pressure was applied until rupture, and the plunger displacement was recorded. Each test was repeated three times for qualitative analysis. Mass transfer performance was evaluated by immersing beads in blue ink for 10 s, followed by washing to remove residual ink. Penetration depth and color intensity were observed, with each test repeated three times for qualitative assessment.

**Text S2** Determination of mechanical properties of immobilized SRB beads

Qualitative description of the mechanical strength of the spheres was performed and the results are shown in Table S7. The optimal level combinations of the factors were 4% for SA concentration, 3% for CaCl_2_ concentration, and 30% for SRB bacterial solution concentration. The primary and secondary order of the effect of each factor on mechanical strength was: SA concentration > CaCl_2_ concentration > SRB bacterial solution concentration. (Chang and Chou, 2002) showed that the higher concentration of sodium alginate would result in larger particle size and stronger mechanical strength, which in turn hindered the entry of external SO_4_^2-^ into the interior of the spheres, and at the same time the internally generated H_2_S could not diffuse out in a timely manner, resulting in the reduction of diffusion coefficient of the sphere matrix, and the death of immobilized bacteria due to nutrient deficiencies and H_2_S toxicity, which would lead to the formation of a death cavity inside the spheres. To satisfy the material exchange between the interior of the spheres and the external environment, it is necessary to choose the appropriate mechanical strength. Therefore, in this study, for the mechanical strength index, the optimal preparation conditions were 2% sodium alginate, 2% CaCl_2_ concentration, and 30% SRB bacterial liquid concentration and 2% sodium alginate, 3% CaCl_2_ concentration, and 40% SRB bacterial liquid concentration. Under these two preparation conditions, after SRB propagation for a period of time, the pellets began to soften and break easily due to mechanical strength, and then began to release the propagated SRB, which led to rapid growth of SRB and promoted the reduction of SO_4_^2-^.

Text S3 Effect of composites on SRB catalase activity and viable cell count (MPN)

At a concentration of 0.1%, the composite materials promoted the growth of SRB. However, when the concentration was increased to 0.5%, SRB growth was inhibited by day 5 (Table S9). These results indicate that at 0.1% concentration, the composite materials did not suppress catalase (CAT) enzyme activity in the SRB culture. In contrast, at 0.5% concentration, certain materials inhibited CAT activity, with a more pronounced and statistically significant inhibitory effect on day 5 compared to day 3. As shown in Table S9, when the composite material was applied at 0.1% (w/v), the viable cell counts of CK, BC, and nZVI@BC on day 3 were 1.2×10^8^, 3.0×10^9^ and 7.0×10^9^ CFU/mL, respectively, and on day 5 were 3.0×10^8^, 2.5×10^9^ and 3.0×10^9^ CFU/mL, respectively. In both time points, the addition of composite materials resulted in higher viable cell counts compared to CK, with nZVI@BC showing the highest bacterial count. At 0.5% concentration, the viable cell counts of CK, 5BC, and 5nZVI@BC on day 3 were 1.2×10^8^, 1.7×10^9^ and 2.0×10^7^ CFU/mL respectively, while on day 5 they were 3.0×10^8^, 7.0×10^8^ and 2.5×10^8^ CFU/mL, respectively. Compared to the 0.1% concentration, bacterial counts for all materials decreased at the same time points. These findings suggest that a 0.1% concentration of composite materials enhances SRB viability more effectively than a 0.5% concentration.

Analysis of CAT enzyme activity and most probable number (MPN) of viable cells indicates that at a 0.1% (w/v) concentration, the composite materials promote SRB growth more effectively than at 0.5%. BC, as an alkaline material, provides adsorption sites favorable for bacterial growth. When added in appropriate amounts, BC creates a pH-buffered environment conducive to SRB proliferation, increasing bacterial density. However, excessive BC raises the solution pH, which becomes detrimental to SRB growth. nZVI@BC contains nZVI, which exhibits reductive properties. During oxidation, nZVI consumes oxygen from the environment, lowering the redox potential of the liquid phase and thereby stimulating SRB growth. Consequently, at a 0.1% concentration, the composite materials effectively enhance SRB proliferation.

Table S1 Composition of improved Starkey medium

| Component | Dosage |
| --- | --- |
| NH_4_Cl | 1.0 g/L |
| Na_2_SO_4_ | 1.0 g/L |
| K_2_HPO_4_ | 0.5 g/L |
| CaCl_2_·2H_2_O | 0.1 g/L |
| MgSO_4_·7H_2_O | 2.0 g/L |
| C₃H₅NaO₃ | 2.0 g/L |
| Yeast extract | 1.0 g/L |
| FeSO_4_·7H_2_O | 0.5 g/L |
| C_6_H_8_O_6_ | 0.1 g/L |
| C_2_H_5_NaO_2_S | 0.1 g/L |
| pH | 7.00 |

Table S2 Orthogonal design table

| Number | A：SA%  （w/v） | B：CaCl_2_%  （w/v） | C：bacterial solution %  （v/v） |
| --- | --- | --- | --- |
| 1 | 2 | 1 | 20 |
| 2 | 2 | 2 | 30 |
| 3 | 2 | 3 | 40 |
| 4 | 3 | 1 | 30 |
| 5 | 3 | 2 | 40 |
| 6 | 3 | 3 | 20 |
| 7 | 4 | 1 | 40 |
| 8 | 4 | 2 | 20 |
| 9 | 4 | 3 | 30 |

**Table S3** Effect of composite materials on the growth of SRB

| Treatments | Material | Dosage%(w/v) | Sampling time |
| --- | --- | --- | --- |
| CK | **/** | **/** | 3 d、5 d |
| BC | BC | 0.1 | 3 d、5 d |
| 5BC | BC | 0.5 | 3 d、5 d |
| nZVI@BC | nZVI@BC | 0.1 | 3 d、5 d |
| 5nZVI@BC | nZVI@BC | 0.5 | 3 d、5 d |

**Table S4** Physicochemical properties of tested soils

| **Soil properties** | **DK soil** | **SF soil** |
| --- | --- | --- |
| pH  Total Cd (mg/kg)  Total Pb (mg/kg)  Total Sb (mg/kg)  Soil organic matter (g/kg)  Alkali-hydrolyzable nitrogen (mg/kg)  Available phosphorus (mg/kg)  Available potassium (mg/kg) | 6.23±0.028  4.69±1.10  1179.29±127.57  109.46±8.93  14.73±0.25  155.52±6.033  11.45±0.37  77.84±12.34 | 7.44±0.051  17.73±2.05  1887.75±46.46  16.22±1.54  12.95±0.18  224.69±13.40  18.02±0.64  62.30±1.36 |

**Table S5** Analysis of specific surface area of immobilized SRB beads

| Material | Specific surface area (m^2^/g) | Total pore volume  (cm^3^/g) | Average pore size  (nm) |
| --- | --- | --- | --- |
| 1 | 1.332 | 0.0021 | 9.506 |
| 2 | 1.095 | 0.0012 | 5.399 |
| 3 | 0.9797 | 0.0016 | 10.92 |
| 4 | 0.7145 | 0.0009 | 5.714 |
| 5 | 1.558 | 0.0035 | 9.321 |
| 6 | 0.5861 | 0.0011 | 6.787 |
| 7 | 0.4864 | 0.0012 | 14.37 |
| 8 | 0.6147 | 0.0016 | 15.73 |
| 9 | 0.4766 | 0.0011 | 11.14 |

**Table S6** Intuitive analysis table of orthogonal design with mass transfer performance as index

| Number | A: SA%  (w/v) | B: CaCl_2_%  (w/v) | C: bacterial solution%  (v/v) | Grade code | Mass transfer performance |
| --- | --- | --- | --- | --- | --- |
| 1 | 2 | 1 | 20 | 6 | excellent |
| 2 | 2 | 2 | 30 | 6 | excellent |
| 3 | 2 | 3 | 40 | 5 | good |
| 4 | 3 | 1 | 30 | 5 | good |
| 5 | 3 | 2 | 40 | 4 | medium |
| 6 | 3 | 3 | 20 | 3 | medium |
| 7 | 4 | 1 | 40 | 2 | bad |
| 8 | 4 | 2 | 20 | 2 | bad |
| 9 | 4 | 3 | 30 | 1 | bad |
| k_i1_ | 17 | 13 | 11 |  |  |
| k_i2_ | 12 | 12 | 12 | T=34 | T^2^=1156 |
| k_i3_ | 5 | 9 | 11 | P=128.44 | Q_T_=156 |
| K_i1_ | 5.67 | 4.33 | 3.67 | S_T_=27.56 | S_E_=0.22 |
| K_i2_ | 4.00 | 4.00 | 4.00 |  |  |
| K_i3_ | 1.67 | 3.00 | 3.67 |  |  |
| R | 4.00 | 1.33 | 0.33 |  |  |
| k_i1_^2^ | 289 | 169 | 121 |  |  |
| k_i2_^2^ | 144 | 144 | 144 |  |  |
| k_i3_^2^ | 25 | 81 | 121 |  |  |
| Q | 152.67 | 131.33 | 128.67 |  |  |
| S | 24.22 | 2.89 | 0.22 |  |  |
| order of priority | A (SA) >B (CaCl_2_) >C (bacterial solution) | | |  |  |
| optimal combination | A1 (SA: 2%) B1 (CaCl_2_: 1%) C2 (bacterial solution: 30%) | | | |  |

Note: k_i_ represents the sum of the experimental results at the i level of the factor; the average value of the experimental results at the i level of the K_i_ representative factor; R represents the range between the results under this factor; Q represents the average value between the results after k_i_ squared; S represents the sum of squares of the mean square; T represents processing and; P represents the correction coefficient; Q_T_ represents processing square sum; S_T_ represents the total variation; S_E_ represents error.

**Table S7** Orthogonal design intuitive analysis table of ball with mechanical strength as index

| number | A：SA%  (w/v) | | B：CaCl_2_%  (w/v) | | C：bacterial solution %  (v/v) | | Propulsion volume of syringe(mL) | | Mechanical strength | |
| --- | --- | --- | --- | --- | --- | --- | --- | --- | --- | --- |
| 1 | 2 | | 1 | | 20 | | 3.4 | | bad | |
| 2 | 2 | | 2 | | 30 | | 4.4 | | medium | |
| 3 | 2 | | 3 | | 40 | | 4.8 | | medium | |
| 4 | 3 | | 1 | | 30 | | 6.7 | | good | |
| 5 | 3 | | 2 | | 40 | | 7.2 | | good | |
| 6 | 3 | | 3 | | 20 | | 7.5 | | good | |
| 7 | 4 | | 1 | | 40 | | 7.8 | | good | |
| 8 | 4 | | 2 | | 20 | | 8.9 | | excellent | |
| 9 | 4 | | 3 | | 30 | | 9.1 | | excellent | |
| k_i1_ | 12.6 | | 17.9 | | 19.8 | |  | |  | |
| k_i2_ | 21.4 | | 20.5 | | 20.2 | | T=59.8 | | T^2^=3576.04 | |
| k_i3_ | 25.8 | | 21.4 | | 19.8 | | P=397.34 | | Q_T_=429.8 | |
| K_i1_ | 4.20 | | 5.97 | | 6.60 | | S_T_=32.46 | | S_E_=0.11 | |
| K_i2_ | 7.13 | | 6.83 | | 6.73 | |  | |  | |
| K_i3_ | 8.60 | | 7.13 | | 6.60 | |  | |  | |
| R | 4.40 | | 1.17 | | 0.13 | |  | |  | |
| k_i1_^2^ | 158.76 | | 320.41 | | 392.04 | |  | |  | |
| k_i2_^2^ | 457.96 | | 420.25 | | 408.04 | |  | |  | |
| k_i3_^2^ | 665.64 | | 457.96 | | 392.04 | |  | |  | |
| Q | 427.45 | | 399.54 | | 397.37 | |  | |  | |
| S | 30.12 | | 2.20 | | 0.04 | |  | |  | |
| number | | A：SA%  (w/v) | | B：CaCl_2_%  (w/v) | | C：bacterial solution %  (v/v) | | Propulsion volume of syringe(mL) | | Mechanical strength |
| order of priority | | A (SA) > B (CaCl_2_) > C (bacterial solution) | | | | | |  | |  |
| optimal combination | | A3 (SA：4%) B3 (CaCl_2_：3%) C2 (bacterial solution：30%) | | | | | | | |  |

Note: k_i_ represents the sum of the experimental results at the i level of the factor; the average value of the experimental results at the i level of the K_i_ representative factor; R represents the range between the results under this factor; Q represents the average value between the results after k_i_ squared; S represents the sum of squares of the mean square; T represents processing and; P represents the correction coefficient; Q_T_ represents processing square sum; S_T_ represents the total variation; S_E_ represents error.

**Table S8** Visual analysis table of orthogonal design of small balls with SO_4_^2-^ reduction rate

| Number | A:  SA%  (w/v) | B:  CaCl_2_%  (w/v) | C:  bacterial solution %  (w/v) | SO_4_^2-^ reduction  Rate (%) | Sulfate concentration  (mg/L) | ORP  (mv) | Blackened medium  Time (h) |
| --- | --- | --- | --- | --- | --- | --- | --- |
| 1 | 2 | 1 | 20 | 45.15 | 814.88 | -382.00 | 78 |
| 2 | 2 | 2 | 30 | 49.18 | 754.88 | -386.33 | 70 |
| 3 | 2 | 3 | 40 | 42.06 | 860.79 | -385.33 | 70 |
| 4 | 3 | 1 | 30 | 40.46 | 884.54 | -361.67 | 78 |
| 5 | 3 | 2 | 40 | 42.89 | 848.45 | -330.00 | 78 |
| 6 | 3 | 3 | 20 | 33.91 | 981.78 | -323.00 | 78 |
| 7 | 4 | 1 | 40 | 35.06 | 964.80 | -293.67 | 120 |
| 8 | 4 | 2 | 20 | 34.50 | 973.06 | -329.67 | 120 |
| 9 | 4 | 3 | 30 | 31.23 | 1021.71 | -302.33 | 120 |
| k_i1_ | 136.39 | 120.66 | 113.56 |  |  |  |  |
| k_i2_ | 117.26 | 126.57 | 120.87 |  | T=354.43 | T^2^=125623.04 |  |
| k_i3_ | 100.78 | 107.20 | 120.00 |  | P=13958.12 | Q_T_=14246.99 |  |
| K_i1_ | 45.46 | 40.22 | 37.85 |  | S_T_=288.88 | S_E_=0.84 |  |
| K_i2_ | 39.09 | 42.19 | 40.29 |  |  |  |  |
| K_i3_ | 33.59 | 35.73 | 40.00 |  |  |  |  |
| R | 11.87 | 6.46 | 2.44 |  |  |  |  |
| k_i1_^2^ | 18601.33 | 14559.66 | 12895.87 |  |  |  |  |
| k_i2_^2^ | 13750.68 | 16020.80 | 14609.56 |  |  |  |  |
| k_i3_^2^ | 10157.29 | 11491.13 | 14400.82 |  |  |  |  |
| Q | 14169.77 | 14023.86 | 13968.75 |  |  |  |  |
| S | 211.65 | 65.75 | 10.63 |  |  |  |  |
| order of priority | A (SA) >B (CaCl_2_) >C (bacterial solution) | | | | |  |  |
| optimal combination | A1 (SA: 2%) B1 (CaCl_2_: 2%) C2 (bacterial solution: 30%) | | | | | |  |

Note: k_i_ represents the sum of the experimental results at the i level of the factor; the average value of the experimental results at the i level of the K_i_ representative factor; R represents the range between the results under this factor; Q represents the average value between the results after k_i_ squared; S represents the sum of squares of the mean square; T represents processing and; P represents the correction coefficient; Q_T_ represents processing square sum; S_T_ represents the total variation; S_E_ represents error.

Table S9 Effect of composites on SRB catalase activity and viable cell count (MPN)

| Treatments | CAT (U/mg) | | The number of live bacteria (CFU/mL) | |
| --- | --- | --- | --- | --- |
|  | Day 3 | Day 5 | Day 3 | Day 5 |
| CK | 7.5796 | 7.7012 | 1.2×10^8^ | 3.0×10^8^ |
| BC | 7.6024 | 8.0343 | 3.0×10^9^ | 2.5×10^9^ |
| 5BC | 7.5773 | 6.8100 | 1.7×10^9^ | 7.0×10^8^ |
| nZVI@BC | 8.2877 | 8.6760 | 7.0×10^9^ | 3.0×10^9^ |
| 5 nZVI@BC | 7.3698 | 6.8892 | 2.0×10^7^ | 2.5×10^8^ |

Table S10 Response of microbial diversity under different treatments

| Soil | Treatment | ace | Chao1 | shannon |
| --- | --- | --- | --- | --- |
|  | SSBC-ZVI  Med  CK | 4228.841±208.0839  3605.685±214.6092  3920.71±247.9687 | 3344.049±167.6203  2857.301±229.3828  3156.419±137.5462 | 5.760857±0.0669  5.21208±0.4485  5.637107±0.0395 |
| DK |  |  |  |  |
|  |  |  |  |  |
|  | SSBC-ZVI  Med  CK | 5331.774±167.5507  3986.407±314.7918  4141.579±179.4982 | 3940.151±100.3351  3057.24±283.7058  3193.059±132.1210 | 6.118497±0.0863  5.23025±0.4156  5.37158±0.3649 |
| SF |  |  |  |  |
|  |  |  |  |  |

Fig. S1 SEM image of SRB.


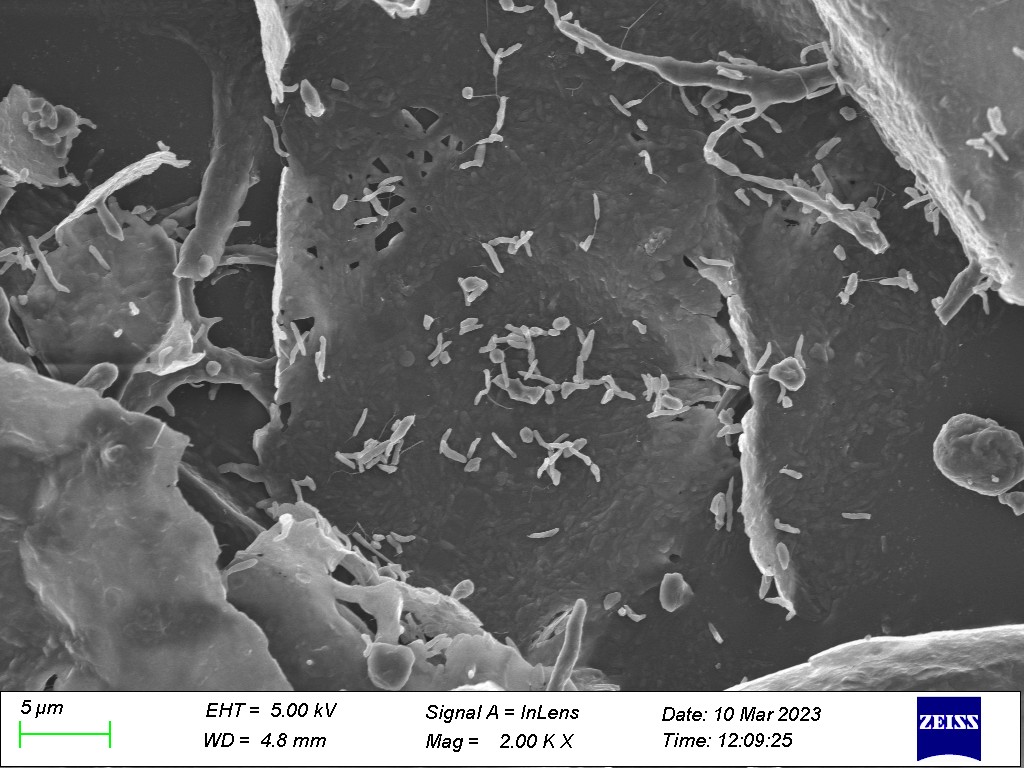


Fig. S2 The changes of SRB at different inoculation amount (A), initial pH (B), and temperatures (C).


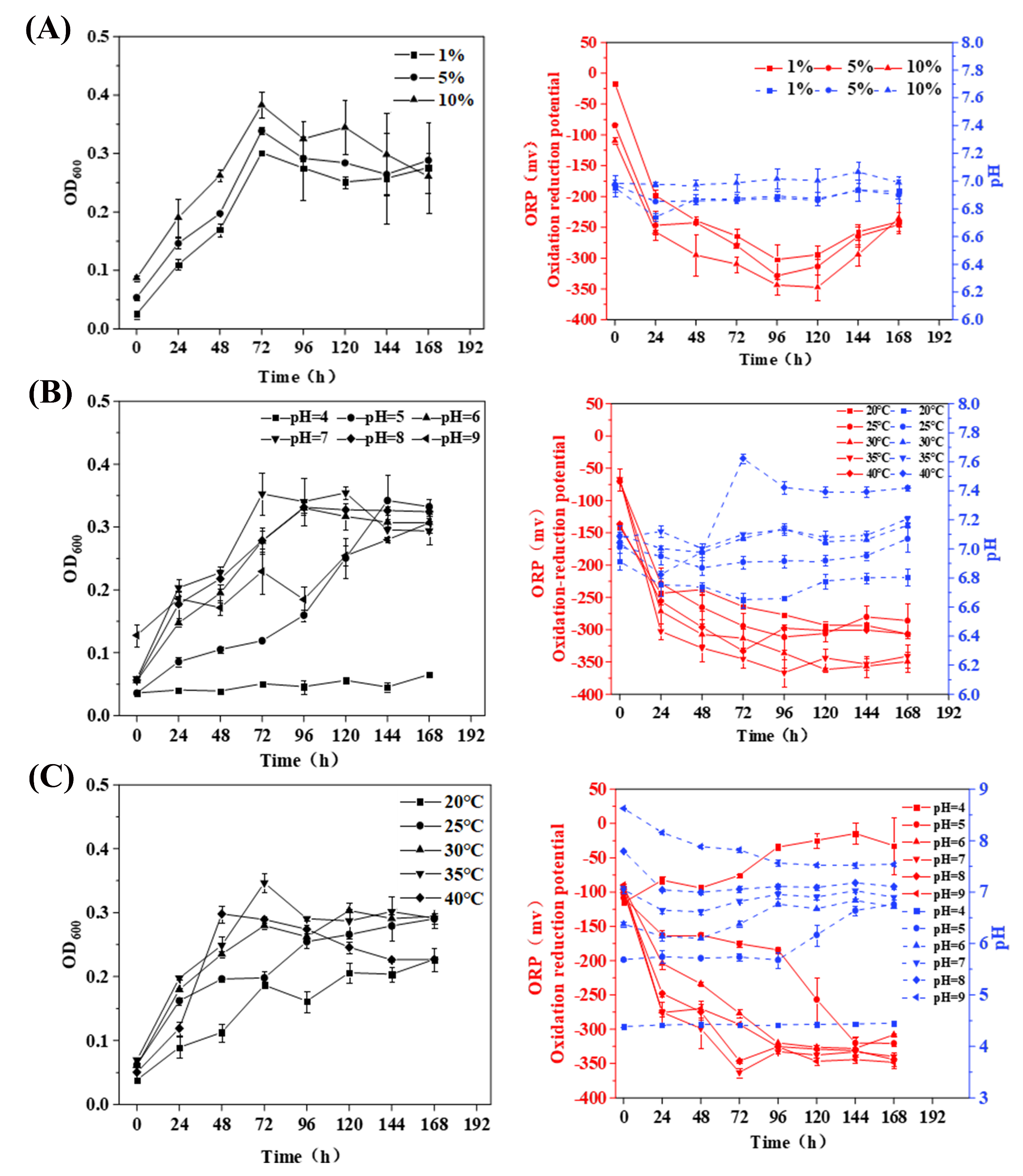


**Fig. S3** Morphology of immobilized SRB beads (A), adsorption-desorption isotherms of different immobilized SRB beads (B), formation of FeS under the cultivation of immobilized SRB beads (C) and morphology of immobilized SRB beads (D).


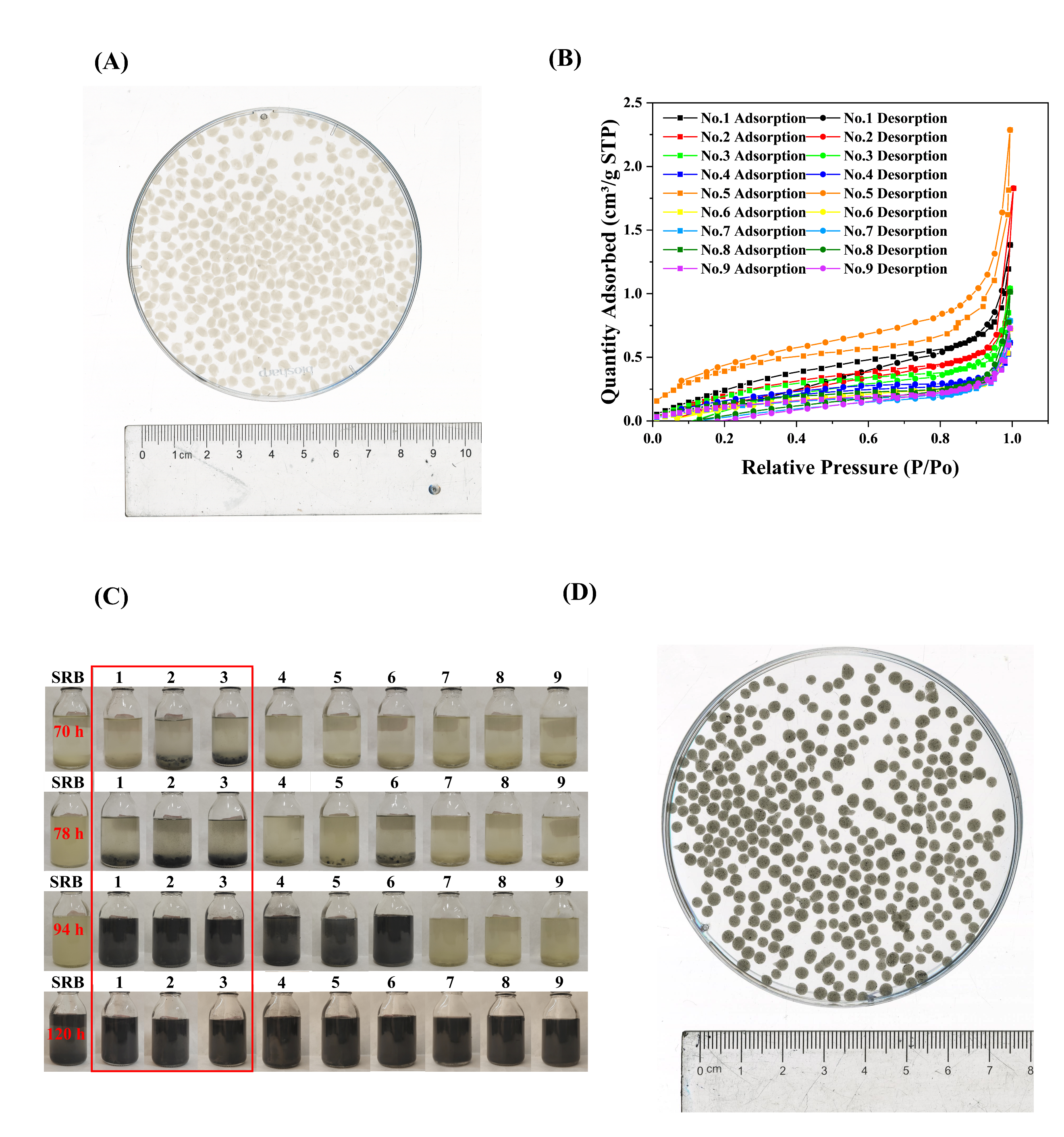


Fig. S4 Determination of mass transfer properties of immobilized SRB spheres.


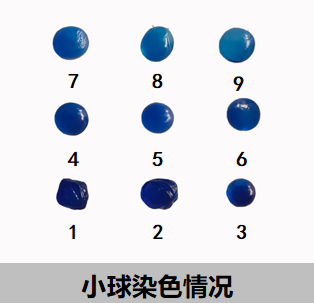


Fig. S5 Production of FeS by three immobilized SRB beads.


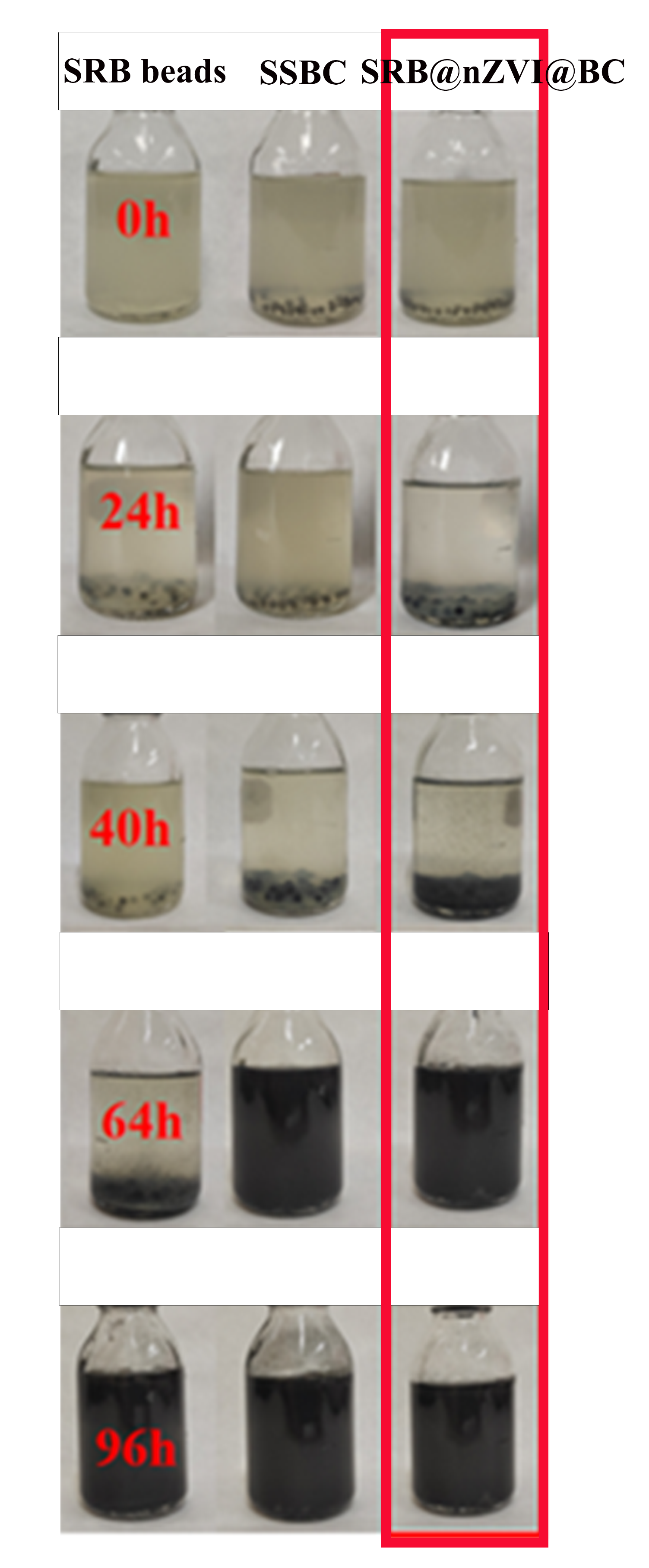


Fig. S6 Effect of different treatments on the activities of catalase (A), urease (B), acid phosphatase (C) and sucrase (D)in DK and SF soils.


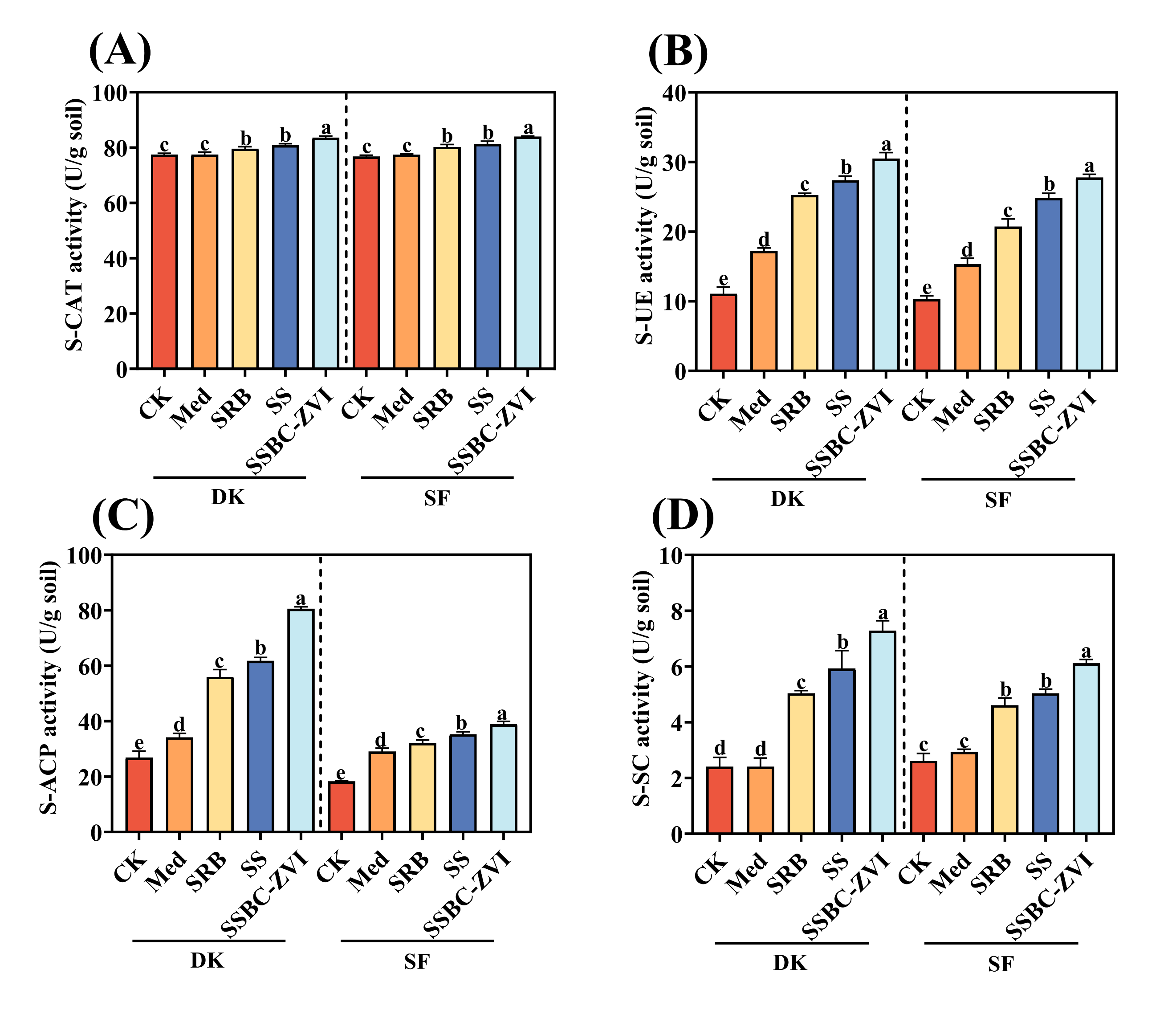


Fig. S7 Effects of different treatments on Cd (A), Pb (B), and Sb (C) concentrations in soil water leachate.


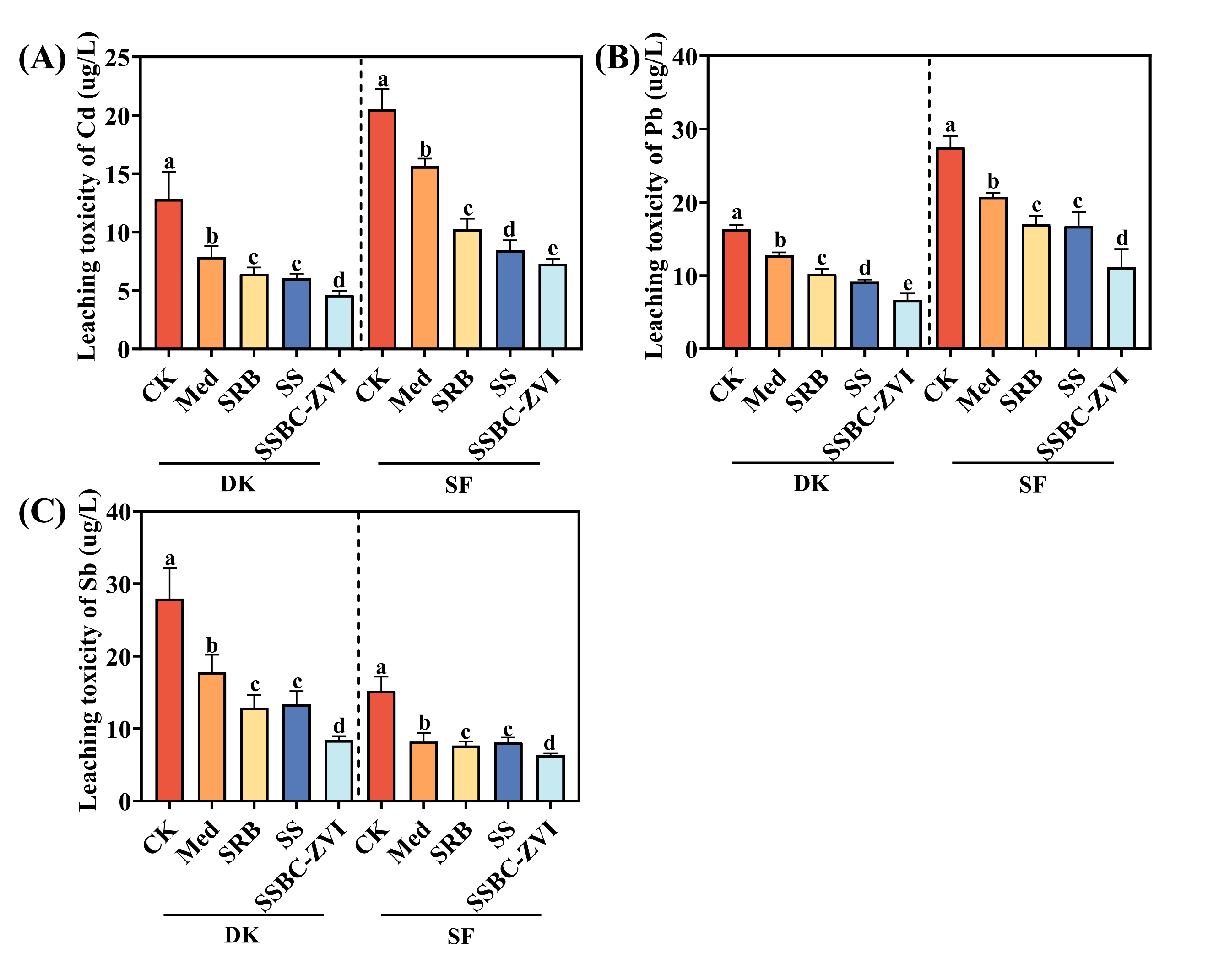


**Fig. S8** FITR (A), XRD (B) and XPS (C) in diffraction pattern analysis of soil particles.
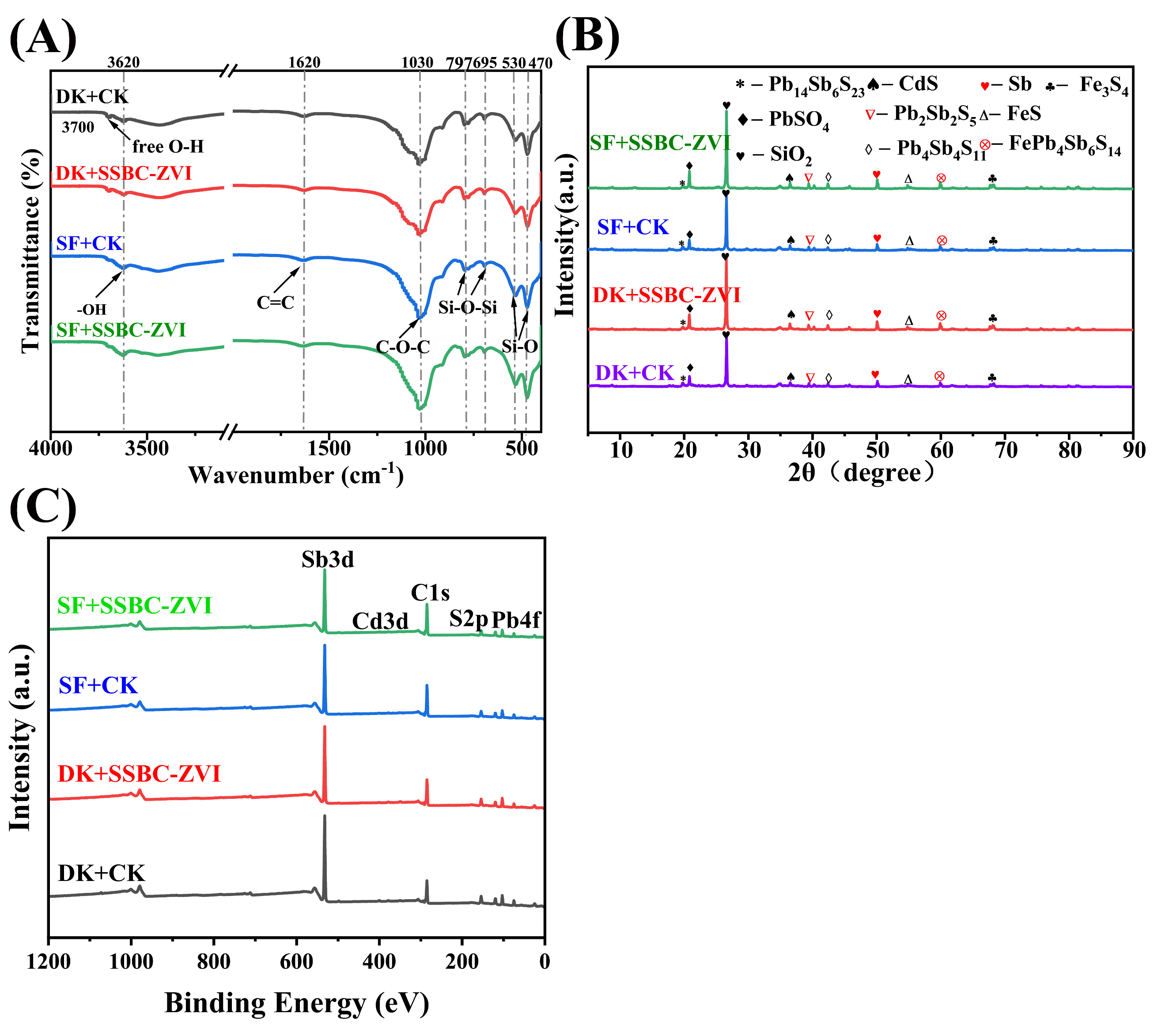


Fig. S9 (A)-(C) are XPS spectra of different adsorbent materials: (A) C1 s, (B) S 2p, and (C) Fe 2p.


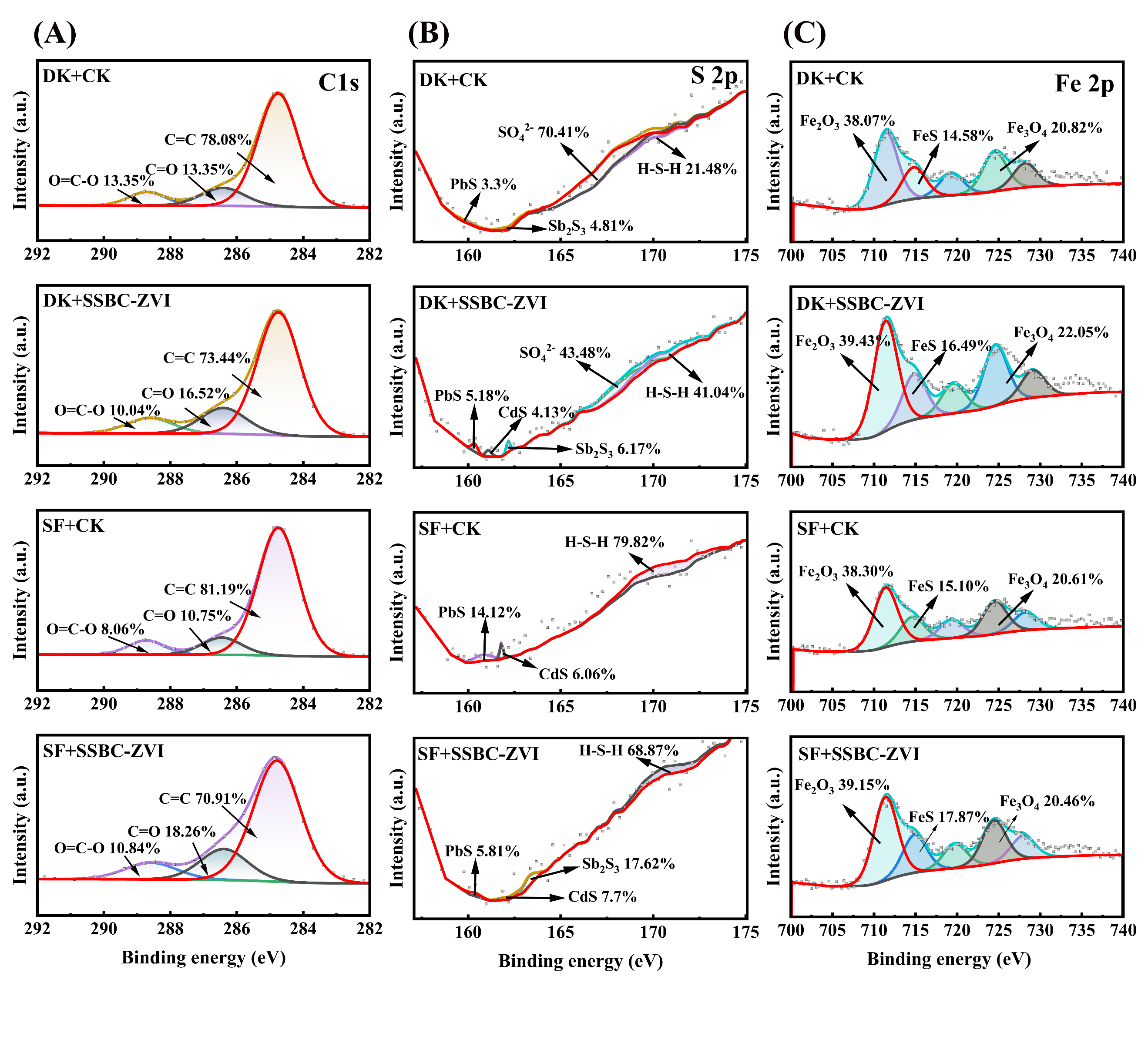


Fig. S10 FITR of nZVI@BC and SRB@nZVI@BC.


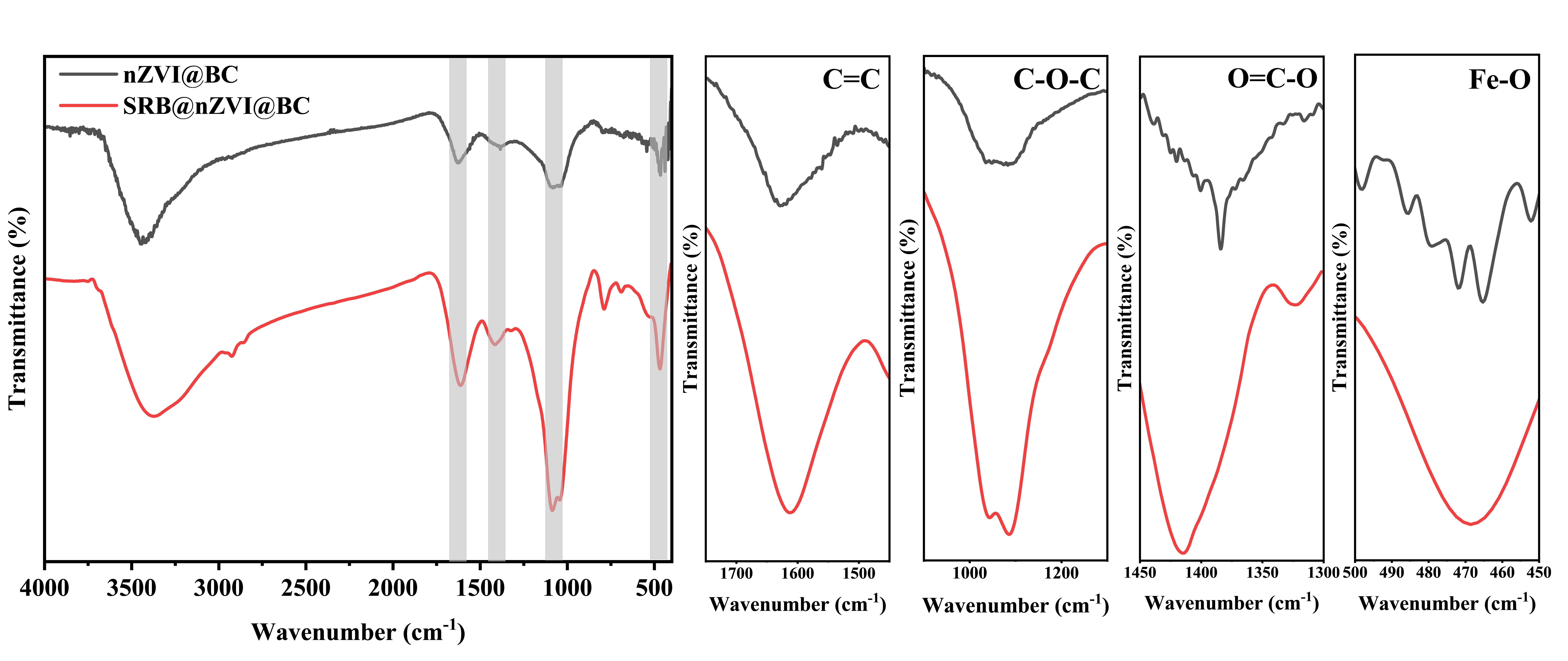


Fig. S11 Structural Equation Model illustrating the effects of soil properties (environment) and SRB on the oxidation of Cd, Pb, and Sb. The paths with insignificant coefficients are not displayed; orange and blue arrows indicate positive correlations, and dashed lines represent negative correlations.


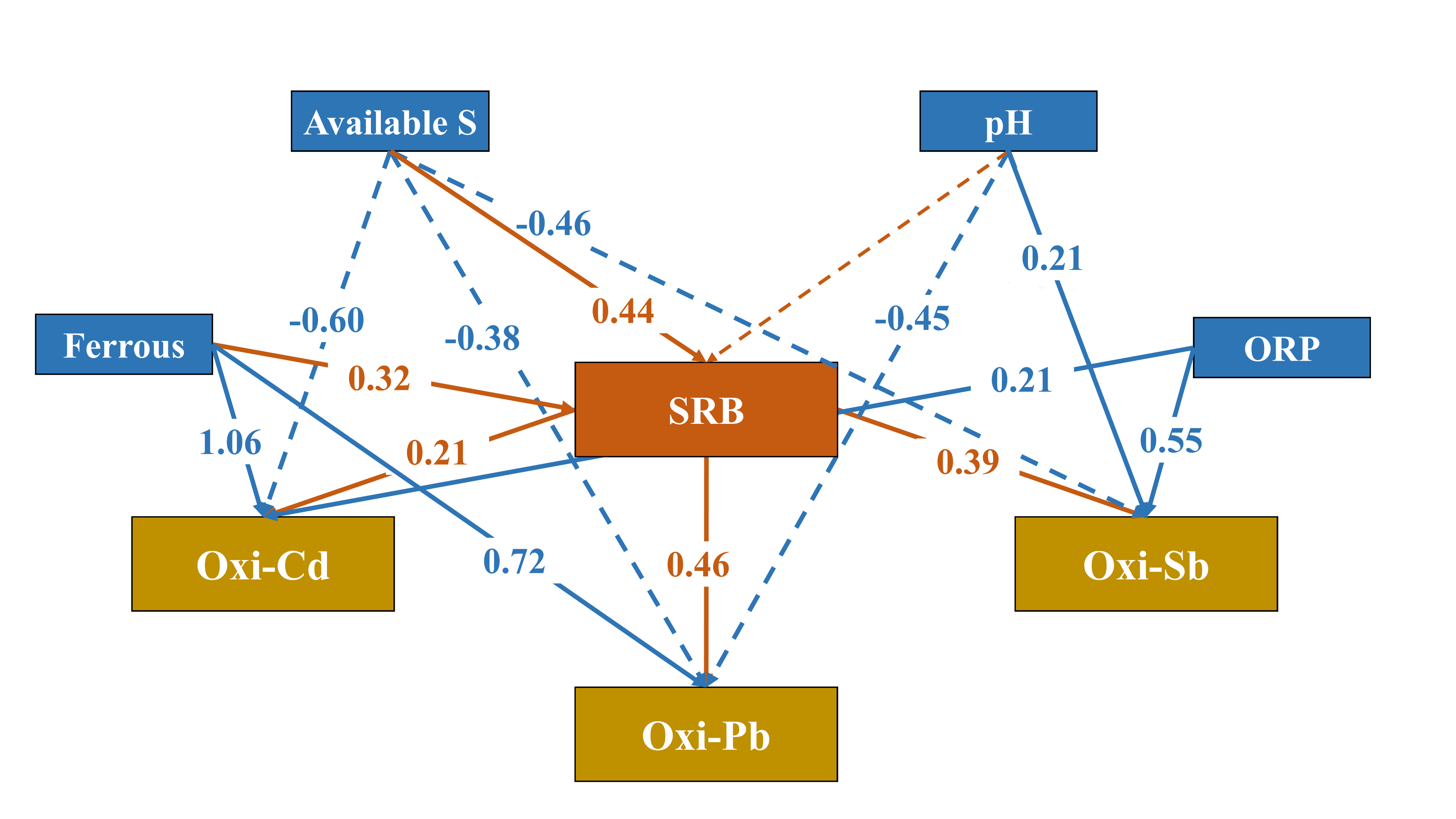


Reference

Chang, Y.C., Chou, C.C., 2002. Growth and production of cholesterol oxidase by alginate-immobilized cells of *Rhodococcus equi* No. 23. Biotechnology and Applied Biochemistry 35, 69-74.<https://doi.org/10.1042/ba20010058>.
